# Skin physiology in microgravity: a 3-month stay aboard ISS induces dermal atrophy and affects cutaneous muscle and hair follicles cycling in mice

**DOI:** 10.1038/npjmgrav.2015.2

**Published:** 2015-05-27

**Authors:** Thibaut Neutelings, Betty V Nusgens, Yi Liu, Sara Tavella, Alessandra Ruggiu, Ranieri Cancedda, Maude Gabriel, Alain Colige, Charles Lambert

**Affiliations:** 1 Laboratory of Connective Tissues Biology, GIGA-Research, University of Liège, Sart Tilman, Belgium; 2 Department of Experimental Medicine, University of Genova, Genova, Italy

## Abstract

**Aims:**

The Mice Drawer System (MDS) Tissue Sharing program was the longest rodent space mission ever performed. It provided 20 research teams with organs and tissues collected from mice having spent 3 months on the International Space Station (ISS). Our participation to this experiment aimed at investigating the impact of such prolonged exposure to extreme space conditions on mouse skin physiology.

**Methods:**

Mice were maintained in the MDS for 91 days aboard ISS (space group (S)). Skin specimens were collected shortly after landing for morphometric, biochemical, and transcriptomic analyses. An exact replicate of the experiment in the MDS was performed on ground (ground group (G)).

**Results:**

A significant reduction of dermal thickness (−15%, *P*=0.05) was observed in S mice accompanied by an increased newly synthetized procollagen (+42%, *P*=0.03), likely reflecting an increased collagen turnover. Transcriptomic data suggested that the dermal atrophy might be related to an early degradation of defective newly formed procollagen molecules. Interestingly, numerous hair follicles in growing anagen phase were observed in the three S mice, validated by a high expression of specific hair follicles genes, while only one mouse in the G controls showed growing hairs. By microarray analysis of whole thickness skin, we observed a significant modulation of 434 genes in S versus G mice. A large proportion of the upregulated transcripts encoded proteins related to striated muscle homeostasis.

**Conclusions:**

These data suggest that a prolonged exposure to space conditions may induce skin atrophy, deregulate hair follicle cycle, and markedly affect the transcriptomic repertoire of the cutaneous striated muscle panniculus carnosus.

## Introduction

Weightlessness, as experienced by astronauts during space flights, affects physiological functions of the human organism that has evolved, like other organisms living on Earth, through continuous adaptation to the permanent gravitational field. Adaptation to reduced gravity implies first body fluids redistribution and unloading of weight-bearing bones and postural muscles. During long-term missions, astronauts suffer from osteopenia due to an increased osteoclasts-mediated bone resorption and decreased formation.^1^ Muscles are also strongly affected by reduced loading demands in weightlessness.^2^ Similar bone loss and muscle atrophy have been observed in rodents maintained in microgravity,^3^ making an acceptable animal model for investigating the mechanisms underlying the space-related health alterations in human.

Besides additional medical problems such as reduced immunity, cardiovascular deconditioning, sensorimotor symptoms, and renal stones formation,^4^ cutaneous alterations represent another major concern for astronauts. A study on 19 crew members of 6 NASA-Mir missions, from 1995 to 1998, indicated that small cutaneous injuries were the most frequent medical incidents.^5^ Recorded complaints include skin dryness and itching making it more susceptible to scratches and irritation. As evaluated by non-invasive procedures on the German astronaut Thomas Reiter before and after a 6-month mission on the International Space Station (ISS),^6^ several cutaneous physiological changes were recorded after the mission such as coarsening of the epidermis and decreased skin elasticity. The most significant change was a thinning of the dermal matrix appearing as low-echo zones on ultrasound images similarly to the skin atrophy observed in aging on Earth. These observations were, however, limited to one test subject. A recent report describes changes of skin sensory input from the foot soles in astronauts following short-duration space flight.^7^ Although playing a vital barrier function against deleterious environmental factors and fluid diffusion and a significant role in thermoregulation and tactile sense, the effect of space conditions on skin physiology has been little investigated.

We had the opportunity to participate in the Mice Drawer System (MDS) Tissue Sharing program,^8^ which was the longest duration animal experiment in orbit (3 months), corresponding to several years in human life, for investigating its impact on mouse skin physiology by morphological, biochemical, and genome-wide analyses. Although the number of available mice for this study was unfortunately small, significant alterations affecting the dermal, hair follicles, and muscular compartments of the skin were observed in these mice as compared with replicate ground experiment.

## Materials and methods

### Mice and MDS spaceflight mission

The MDS experiment was approved by the American Institutional Animal Care and Use Committee (IACUC protocol n° FLT-09-070-KSC) and performed in accordance with the principles expressed in the NIH ‘Guide for the care and use of laboratory animals’ and following recommendations reported in European Communities Council Directive of 24 November 1986.

The experiments were carried out using 8-week-old, at the time of the launch, male C57Bl/J10 wild-type (WT) mice and transgenic (Tg) mice of the same lineage overexpressing the osteogenic factor pleiotrophin/osteoblast stimulating factor 1 (PTN/OSF1) under the control of the human bone-specific osteocalcin promoter. These Tg mice were used by the initiator and principal investigator of this program (RC) to investigate a potential protection against space-induced osteoporosis.^9^

The Italian Space Agency appointed Alcatel–Alenia Space to develop the MDS, a payload designed for long duration rodent research on ISS.^10^ This facility can function autonomously for >3 months with minimal maintenance activities by the crew. It provides six mice in individual housing with a controlled delivery of food and water, ventilation, and light. It also includes six cameras to check for health status and behavioral observations of the mice. A detailed description of the MDS is given in ref 8 and at http://www.nasa.gov/mission_pages/station/research/experiments/665.html.

An international ‘Tissue Sharing Program’ gathering 20 research groups from 6 countries was organized by Italian Space Agency in collaboration with ESA, NASA, Japanese, and Canadian Space Agencies to obtain a maximum of data from this unique experiment. Three WT and three Tg mice were individually housed in the MDS (space group (**S**)) that was integrated in the middeck of the shuttle Discovery, flight STS-128, to reach the ISS on 28 August 2009 and transferred to the Express Rack 4 in the Japanese Experiment Model onboard of ISS until return to Earth by the shuttle Atlantis (STS-129) on 27 November 2009. On total, mice spent 91 days in weightlessness which is the longest duration animal experimentation in space. Mice were provided with water *ad libitum* and 5 g of dry food per day. During the 3-month period on the ISS, one Tg and two WT mice unfortunately died and were stored at −20 °C for performing post-flight analysis of bones as described.^9^ Less than 3 h after landing on Earth, the three remaining living mice were transported to the Life Support Facility at KSC where body weighing and collection of urine and blood were performed before sacrifice by carbon dioxide inhalation followed by dissection, collection, and processing of the different tissues and organs. TN, BVN, AC, and CL were not directly involved in the design and execution of the experiment. The collection of skin samples was performed by YL, ST, and AR under the supervision of RC according to a predesigned protocol agreed by all authors. A detailed description of pre-flight, on orbit and post-landing operations, as well as data on animal behavior, and weight gain has been previously published.^8^

A ground control experiment (ground group (G)) replicating food and water supplementation and environmental conditions was conducted in a MDS from 13 November 2009 to 11 February 2010 in the animal facility of the Advanced Biotechnology Center at the University of Genova. One Tg and two WT mice were sacrificed on the same experimental day as the corresponding space mouse death and placed at −20 °C. The body weight gain was similar in the G and S group (G: +5.8±1.4 g; S: +5.5±0.9 g).^8^

### Skin samples collection

After roughly shaving hair, skin specimens of ~1.5 cm^2^ were collected in the head and tail regions on either dorso-lateral side of the body (four skin samples per mouse) and snap frozen in liquid nitrogen before storage at −80 °C and transportation in dry ice to our laboratory in Liège (Belgium). A transverse fragment in the middle of the body was fixed in formalin for 2 h, transferred to 70% ethanol, transported at room temperature, and stored at 8 °C until processing for histological analysis.

### Histological, immunochemical, and histomorphometric analyses

Sections (5 μm) from full thickness skin pieces collected in four zones of the sample dedicated to histological analysis were stained with hematoxylin & eosin and Masson’s Trichrome. Immunostaining of blood vessels was performed with anti-CD31 antibody (#DIA310, Dianova GmbH, Hamburg, Germany) and revealed with a rabbit anti-rat biotinylated conjugated secondary antibody (#E0468) and streptavidin/horse radish peroxidase (#P0397) both purchased from Dako (Golstrup, Denmark).

The thickness of the dermis, hypodermis, and panniculus carnosus was measured on four hematoxylin & eosin-stained sections from each mouse at eight different locations of the section by image analysis using ImageJ software (NIH, Bethesda, MD, USA). The area occupied by CD31-positive vessels was measured in the three cutaneous layers (dermis, hypodermis, and panniculus carnosus) using ImageJ and expressed in percentages of the total field surface. The number of growing hair follicles^11^ was counted in four different skin sections of each mouse and expressed per unit length of epidermis.

### Skin collagen content and extractibility, and skin hydration

Two full thickness skin fragments of ±1 mm^2^ were sampled from each of the four dorso-lateral pieces of the tissue collected in the 3S and 3G mice (*n*=8 per mouse) and weighed (wet weight). After hydrolysis in 6 N HCl for 3 h at 138 °C, collagen content was determined by the colorimetric assay of hydroxyproline.^12^

Full thickness skin fragments of about 100 mg were collected in duplicate from the same four dorso-lateral pieces of tissue in the 3S and 3G mice and weighed before and after lyophilization. The water content was calculated as the difference between wet and dry weight. The lyophilized samples were powdered at liquid nitrogen temperature in a Mikro-Dismembrator S (Braun Biotech International, Melsungen, Germany) and sequentially extracted in 20× volume of 0.15 M NaCl, 0.05 M Tris-HCl, pH 7.4 containing a cocktail of proteases inhibitors followed by 1 M NaCl, 0.05 M Tris-HCl, pH 7.4 and then by 0.5 M acetic acid brought to pH 2.0 with HCl, each extraction being performed for 48 h at 4 °C and followed by a centrifugation at 10,000 r.p.m. at 4 °C. The solubilized collagen was measured in each supernatant by hydroxyproline assay as above.

### RNA preparation, microarray analysis, and real-time RT–PCR

RNA was prepared from individual S and G skin samples from the head and tail regions using RiboPure kit (Ambion, Austin, TX, USA) according to the manufacturer’s instructions. All RNA samples had a RNA quality indicator ⩾7.6 as assessed by capillary electrophoresis (Agilent 2100 Bioanalyzer, Agilent Technologies, Santa Clara, CA, USA). Complementary DNA was hybridized to Genechip Mouse Genome 430 2.0 arrays (Affymetrix, Santa Clara, CA, USA) at the Genomic Platform of the GIGA (University of Liège, Belgium). Only transcripts having a signal level above 100 U in at least one sample were considered as expressed.

Microarray data are available in the ArrayExpress database (www.ebi.ac.uk/arrayexpress) under accession number E-MTAB-2871.

The expression of selected transcripts was validated by real-time reverse transcription–PCR (RT–PCR) using appropriate primer pairs (Supplementary Table 1S) giving amplicons of expected size as assessed by end-point RT–PCR and gel electrophoresis. RNA (1 μg) was reverse transcribed using a SuperScript III kit (Invitrogen #18080-044, Carlsbad, CA, USA) and oligo-dT primer. Amplification was made on a quantity of complementary DNA corresponding to 10 ng of RNA, appropriate primer pairs (Supplementary Table 1S), and a quantitative PCR Mastermix Plus for SYBR Green I—dTTP kit (# RT–SN2X-03+WOUN; Eurogentec, Liège, Belgium) as described by the manufacturer in a real-time thermocycler (Applied Biosystems 7300, Life Technologies, Gent, Belgium). After 10 min of denaturation at 95 °C, complementary DNA was amplified for 40 cycles (95 °C for 10 s, 60 °C for 1 min). A melting curve performed at the end of each run indicated that the amplification product had a single melting temperature, suggesting that no primer dimers were formed. The efficiency of the amplification was calculated by the serial dilution method. Calculation of the transcript expression level was made using the Cq of the genes-of-interest and of the calibrators (GAPDH, β-actin, and β2-microglobulin) and the ΔΔCq method.

## Results

### Experimental groups and statistical analysis

From the six mice transported to the ISS, three of them (two WT and one Tg) unfortunately died during the mission and were cryo-preserved at −20 °C onboard the ISS. The post-landing necropsy revealed that one animal died from a major spinal cord injury that likely occurred during shuttle lift off, the second one possibly from liver pathology, and the third one due to a failure of the food delivery system of the MDS. The remaining three living mice showed a normal behavior throughout the entire mission and appeared in excellent health conditions at landing.^8^ The ground control experiment was designed to repeat exactly the events that occurred during the on-orbit mission. As the leading experimenter (RC) could use frozen bones for computed microtomography analysis, two WT and one Tg mice from the G group were killed and frozen at the same experiment day as the lost mice on ISS. Hence, one WT and two Tg animals in each group were available for our investigations.

**Figure 1:**
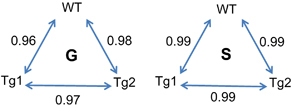
Pair-wise Pearson’s correlation rates (*r*^2^) between individual transcripts levels analyzed by microarray in wild-type (WT) and transgenic (Tg) mice in ground (G) and space (S) groups.

We are well aware that the small number of available experimental animals in both groups, that further contain one WT and two Tg mice, is a limiting factor to the power of statistical analysis. Our first concern was therefore to evaluate the expression of PTN/OSF1 relative to the housekeeping gene *GAPDH* in the skin of the two genotypes. As measured by quantitative RT–PCR, the normalized expression of PTN/OSF1 was similar in Tg (1.03±0.12) and WT mice (1.23+0.35) as also confirmed by microarray data (Tg: 5578±1119; WT: 6309+1773), indicating that the *PTN/OSF1* gene was not overexpressed in the skin of Tg mice. This result was expected, since this transgene was driven by a bone-specific promoter. As additional validation, we used raw data from the microarray interrogating ~49,700 transcripts to compare the three mice within their own group, G or S, by establishing pair-wise Pearson’s correlation rates between the individual levels of expressed transcripts (Figure 1). The high correlation rates indicated that the WT and the two Tg mice were not significantly different from each other within their group authorizing us to group the two genotypes for comparing data from the three mice in the G and S groups.

### Skin histology and histomorphometry

**Figure 2:**
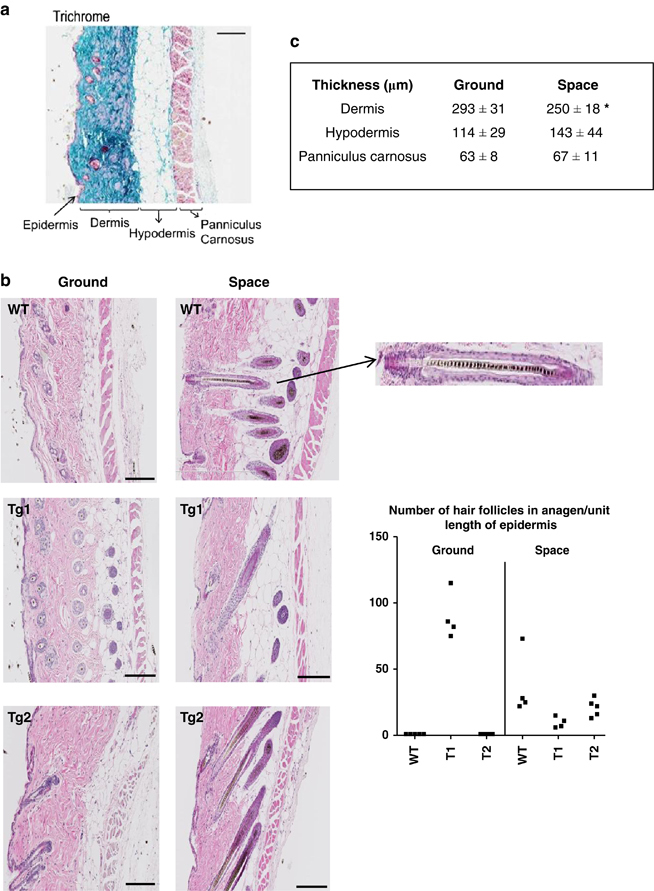
Skin histology. (**a**) Masson’s trichrome staining of a ground control mouse showing the different skin layers. (**b**) Hematoxylin & eosin staining of full thickness skin sections of the three mice of the ground and the space group (WT, Tg1 & Tg2); the arrow points to an enlarged hair follicle showing the melanin granules typical of growing hair in anagen phase; the right panel illustrates the number of growing hair follicles penetrating the hypodermis per unit length of epidermis in the ground and space group. (**c**) Mean thickness in micrometer of the dermis, hypodermis, and panniculus carnosus of the skin in the ground and space group. *P=0.05, Student’s *t*-test. Bar=200 μm.

Masson’s Trichrome staining in Figure 2a shows the murine skin structure comprising a thin epidermis made of two to three layers of keratinocytes, a collagen-rich dermis, a layer of adipose tissue (hypodermis), and a lamina of striated muscle (panniculus carnosus). As seen by hematoxylin & eosin staining (Figure 2b), the three S mice displayed a typical pattern of actively growing hair follicles that penetrate the hypodermis down to the panniculus carnosus (anagen stage of the hair follicle cycle).^11^ Melanin granules were clearly visible in the hair shaft, a typical feature of anagen stage, as shown in the enlarged hair follicle of Figure 2b. Only one mouse among the 3 G mice (Figure 2b, Tg1) displayed this pattern. The number of hair follicles in anagen stage recorded for each mouse is detailed in the right panel of Figure 2b.

The thickness of the three cutaneous layers (dermis, hypodermis, and panniculus carnosus) was measured by image analysis. The thickness of the dermis was significantly reduced (−15%, *P*=0.05) in S mice as compared with the G controls (Figure 2c). Although not statistically significant, the hypodermis tended to be thicker in the S mice due to the presence of hair follicles. The panniculus carnosus thickness was similar in both groups. The surface covered by blood vessels was evaluated on CD31-stained sections for each skin compartment. No significant difference was observed between S and G mice (data not shown).

### Skin biochemical parameters

As detailed in Table 1, skin hydration and total collagen content on a weight basis were similar in both groups of animals. A significant increase of newly synthesized procollagen, characterized by its extractibility in neutral saline solutions, was measured in the S group as compared with the G controls. The amount of acid soluble collagen corresponding to older collagen deposited in the dermal extracellular matrix (ECM) was not significantly altered.

### Effect of space conditions on gene expression in skin

A genome-wide analysis was performed on total RNA extracted from the skin of the six individual mice. The use of the ‘Mouse Genome 430 2.0 Affymetrix’ arrays allowed to interrogate over 49,700 transcripts corresponding to ~22,000 well-characterized mouse genes. About 11,000 different transcripts were detected at a significant level (>100 U). Using a cut-off of 2.0 for fold change and a *P*-value ⩽0.05 for statistical significance between S and G mice, we found 434 transcripts consistently differentially expressed in the three S mice versus the three G mice (292 upregulated and 142 downregulated). The full list is provided in Supplementary Table 2S. The expression of a panel of genes commonly used as calibrators was similar in both groups (space versus ground: *Gapdh*: 1.53; *Actb* (β actin): 1.03; *Actg1* (γ actin): 0.93; *Ppia* (cyclophilin A): 0.91; *Ppib* (cyclophilin B): 0.96; *B2m* (β2 microglobulin): 1.02; *tubb1* (β-tubulin): 0.74; *Hprt1* (hypoxanthine guanine phosphoribosyl transferase 1): 0.83; mean fold change=0.98). The expression level of the 434 identified genes was similar for the three mice in their own group whatever their genotype as evaluated by a *χ* ^2^-test (*P*<0.0001 for both groups). RT–quantitative PCR quantifications were performed to further validate some microarray data (Supplementary Table 3S). Significant correlation (Pearson’s correlation coefficient=0.78, slope=0.75) was found between the two analytical techniques, validating the reliability of our microarray-based analyses.

#### Genes involved in ECM homeostasis

Our first approach was to analyze the expression of genes involved in cutaneous ECM homeostasis, such as collagens, proteoglycans, elastic fibers components, matrix metalloproteinases (MMPs), their activators, and inhibitors. The significantly modulated ECM genes, selected by using a cut-off of ⩾1.5 to better evaluate potential subtle changes in cutaneous structural proteins and related enzymes, are shown in Table 2 and some relevant genes were validated by RT–quantitative PCR (Supplementary Table 3S). The full list of ECM expressed genes is provided in Supplementary Table 5S. The α1 chain of type I collagen (*Col1a1)* and two matricellular proteins controlling collagen turn-over, connective tissue growth factor (*Ctgf/CCN2),* and cysteine-rich angiogenic inducer 1 (*Cyr61/CCN1)* were upregulated in S skin. Among the other significantly modulated genes, we pointed out a reduction of prolyl-4 hydroxylase, tenascin X, emilin-2, hyaluronan synthase 2, and an increased expression of enzymes involved in proteoglycans degradation, *Adamts 1 and 9*. The murine interstitial collagenase *Mmp13* was not expressed and none of the other expressed *Mmps* was significantly modulated (Supplementary Table 5S).

#### Genes involved in hair follicles cycling

In agreement with the histological findings shown in Figure 2, the keratins specific of hair follicles growth were strongly expressed in the 3S mice and in the sole G mouse (Tg1) that showed growing hair follicles (Table 3). A significant correlation between the specific hair follicle keratin 25 and the number of hair follicles in anagen as measured in Figure 2b was established (*r* ^2^=0.76, *P*=0.02). Other genes modulated during hair follicle cycle such as keratin-associated proteins were similarly upregulated (not shown). On the opposite, keratins specific of the interfollicular epidermis were expressed in S and G mice at a similar level pointing to a specific effect of space conditions on hair follicles.

#### Genes involved in striated muscle function

Among the differentially expressed transcripts in S mice (⩾2.0), 87 upregulated and 1 downregulated transcripts encode structural muscle proteins or proteins involved in muscular contraction, differentiation, and atrophy, in neuro-muscular junction and excitation–contraction coupling, in glycogen breakdown and glycolysis (Table 4). They included the sarcomeric giant proteins titin and nebulin, and the myosin light chain kinase 2. Other motor proteins, such as skeletal muscle actin, myosin light chain 1, and myosin light chain phosphorylatable fast skeletal muscle were also upregulated in the S mice but at a lower extent (1.5- to 2-fold). Other muscle genes (as *Myh4, Myh9, Myh10, Myh11, Myh14, Myl2, Myl9* and *Acta2*) were not significantly modified, indicating some specificity of the space-related regulations.

Among the transcription factors regulating myogenesis, myocyte enhancer factor 2 (MEF2) and myogenin were upregulated in S mice. A large number of genes highlighted in Table 4 are potential or recognized targets of these two transcription factors. Other genes not directly related to muscle homeostasis but potentially regulated by MEF2C and myogenin and overexpressed in space conditions were also identified (Supplementary Table 4S). It is noteworthy that PAI1 had a fourfold higher expression in the S mice. Altogether 20% of the total number of upregulated genes is directly related to MEF2/myogenin activity and expression.

The most significant enriched pathways, biological processes, and molecular functions, identified by the ToppFun software (http://toppgene.cchmc.org), were all related to muscular system and glycogenolysis (Table 5). Gene set enrichment analysis also revealed that a potential binding site for the transcription factor MEF2 occurs in many of these genes.

## Discussion

The limited number of mice that can be housed in the MDS payload together with the unfortunate loss of three mice, represents a critical aspect of the experiment, especially for the reliability of statistical analysis. It was, however, a unique opportunity to study the effects of a long-term exposure to microgravity on several tissues in an animal model and to collect observations that might be relevant for future investigations. The similar levels of OSF1/PTN expression in the skin of the WT and Tg mice and the highly similar gene expression profile found by transcriptomic analysis in the two genotypes within a given experimental group authorized us to group both strains for consolidating the comparisons between the S and G group.

Skin has not yet received much interest in space research although it is the largest organ of the human body. It has multiple functions including thermal regulation, tactile sense, protection against pathogens and deleterious environmental conditions such as radiations and provides a vital barrier against body fluids diffusion and might be the source of health problems, including carcinogenesis, during long term space travels. The three-month duration exposure to weightlessness conditions experienced by mice during the MDS mission is appreciable and provided 20 research teams with organs and tissues collected from these ‘astromice’. The results published at the present time showed a bone loss in the weight-bearing bones,^9^ atrophy of the antigravity soleus muscle with a concomitant slow-to-fast transition,^13^ modulation of gene expression in the brain,^14^ increased lipid peroxidation products, and anti-oxidant defenses in erythrocytes.^15^ In our study, three major findings concerning different compartments of the skin are reported. Skin is a complex organ made of a stratified keratinizing epithelium, a dermis, and a hypodermis. In mice, it contains a large number of hair follicles, appended sebaceous glands, and arrector pili muscles. Epidermis is separated from the underlying dermis by a dermo-epidermal junction made of a basement membrane supporting the basal layer of germinal keratinocytes. The dermis is made of a scaffold of polymerized fibrillar collagens I, III, and V organized in thick bundles embedded in a highly hydrated gel of hyaluronic acid and proteoglycans. Many adhesive glycoproteins, matricellular proteins, growth factors, and biological mediators are associated with the dermal ECM. This matrix is populated by fibroblasts, which contribute to the maintenance of their own support through a remodeling process which proceeds at a slow rate in healthy adults. Beneath the dermis lies the hypodermis, made of a layer of adipose tissue, and a thin lamina of striated muscle, the panniculus carnosus. Contrasting to muscle and bone cells, dermal fibroblasts are not usually considered as ‘professional’ cells in mechano-sensing and mechano-reaction. We and others have, however, shown *in vitro* that they are mechano-competent and respond to modification of their mechanical environment by a marked regulation of their metabolic activity,^16,17^ including in microgravity.^18^

In the present *in vivo* study, a significant thinning of the dermis was found in the S mice although it was invaded by growing hair follicles, a process that is known to induce an appreciable increase of its thickness.^19^ The skin atrophy as shown here is therefore probably largely underestimated. The reduced dermal thickness and the consequent decreased collagen content per unit surface suggest a progressive degeneration of the dermis in agreement with the echographic data obtained in one astronaut.^6^ Collagen solubilized at low ionic strength at neutral pH represents the newly synthesized procollagen molecules. Its significant increase in S skin suggests an increased turn-over rate. This hypothesis is supported by the upregulation of α1(I) collagen messenger RNA expression and that of two matricellular proteins, CTGF/CCN2 known to stimulate collagen synthesis downstream of TGFβ pathway^20^ and Cyr61/CCN1, which induces a senescent phenotype associated with an increased collagen degradation.^21^ Furthermore, one can speculate that the newly formed procollagen molecules are underhydroxylated, as suggested by the observed reduced expression of prolyl-4-hydroxylase, and more prone to lysozomal intracellular degradation, as seen for instance during vitamin C deficiency.^22^ An impairment of procollagen processing and mature collagen fiber formation might also be involved due to a reduced expression of the enhancer (Pcolce2) of procollagen carboxypeptidase (BMP1). We propose that the dermal atrophy as seen in the S mice might be related to an imbalance between synthesis and degradation with an excessive early degradation of newly formed, perhaps defective, procollagen molecules. Moreover, the reduced expression of hyaluronan synthase, responsible for hyaluronan synthesis coupled to an increased expression of the aggrecanases *Adamts 1* and *9* may further participate in the skin atrophy observed in space environment. It is noteworthy that senescent human skin is characterized by a reduction of hyaluronan.

One surprising finding of this study was the presence of a significant proportion of hair follicles in anagen phase in the three mice in Space. These morphological data were validated by the high expression level of a series of keratins known to be specifically expressed in hair follicles during the anagen phase while the interfollicular epidermal keratins were not modified. Follicle cycle, comprising an anagen, catagen, and resting telogen phase, starts soon after birth and is synchronous for the two first cycles before becoming asynchronous. According to the relative duration of hair cycles in C57BL6 mice,^11^ most hair follicles in these mice, that were 21-week-old at the end of the experiment, should be in resting telogen phase. As the anagen phase normally represents around ¼ of the total cycle duration at this age, it means that the probability to have three mice in anagen stage is 1/64, suggesting that our observations are probably not fortuitous. It might indicate an increase of the relative duration of the anagen phase in microgravity. As hair cycle depends on cyclic activation and silencing of hair stem cells, this suggests that microgravity might alter their differentiation program as observed in several models in microgravity.^23^

Microgravity affects muscles mass and physical performance in humans and animals.^24^ In general, slow type contractile proteins were found to be reduced in microgravity concomitantly to an increase of the fast type ones and of proteins involved in glycolytic activity.^25^ The major finding of the microarray analysis performed in our study highlighted the overexpression in the skin of a high number of structural genes expressed in striated muscles and of genes involved in contraction or encoding transcription factors positively acting in myogenesis or muscle development. As pili arrector muscles and muscle cells lining blood vessels are smooth muscles, the only candidate that could express these specific striated muscle genes is the panniculus carnosus. It is considered as a fast striated muscle as it exhibits fast myosin fiber subtype, is negative for slow myosin and show striations typical of contractile sarcomeres.^26^ This cutaneous muscle has been reported to be a site of exceptionally rapid wound healing and angiogenesis^27^ and is believed to participate in thermoregulation. Furthermore, it displays a huge capacity of homing bone marrow-derived stem cells as compared with other muscles, suggesting its high regenerative capacity.^26^ Increased expression of a number of genes specific of fast type muscle and genes encoding enzymes involved in glycolysis suggests a strengthening of the ‘fast’ phenotype of the panniculus carnosus in microgravity.

Among those genes that are specifically upregulated in the skin of S mice, many encode transcription factors involved in myogenesis such as Myf6, myogenin, MEF2C, and MEF2D, and proteins involved in muscle contraction, neuromuscular junctions, and bioenergetics known or potential targets of MEF2 or myogenin. Together, these data suggest that microgravity increased the expression of a high number of genes in the panniculus carnosus in part through a MEF2C–myogenin pathway.

Muscle atrophy as reported in microgravity may result from both cell death and myofibrillar protein degradation. An increased expression of *Trim63* and *Fbxo32* genes, two striated muscle-specific proteins associated with the ubiquitin proteasome, and of *Serpine1*, a gene encoding PAI-1 was observed in a number of models of muscle atrophy, including microgravity and unweighing.^28,29^ The study performed by Sandona *et al* ^13^ on the mice muscles in this Tissue Sharing Program showed an increased expression of *Trim63* in the soleus, and of *Trim63* and *Fbxo32* in the extensor digitorum longus. However, muscle atrophy was detected only in the slow postural soleus. Here, we also recorded increased levels of *Trim63*, *Fbxo32,* and *Serpine1* messenger RNA in the skin of S mice but the thickness of the panniculus carnosus did not seem to be affected by exposure to microgravity, suggesting that it was not atrophied. It is possible that non-postural muscles, such as the panniculus carnosus and extensor digitorum longus, are less sensitive to microgravity.

A recent publication^30^ showed that many oxidative stress/anti-oxidant defense genes as well as genes encoding ECM structural components were upregulated in the skin of mice flown for 13 days in a space shuttle. None of these reported genes were modulated in our experiment. These discrepancies most probably arise from the very different experimental design, notably in terms of duration of exposure to space environment. Our data may represent a long-term adaptative behavior to microgravity.

Altogether, we have shown that skin may be a target of space flight conditions that lead to dermal atrophy, deregulation of hair cycle, and modulation of the transcriptomic repertoire of the striated muscle panniculus carnosus in mice. This suggests that the skin of astronauts may be affected by pathophysiological alterations that could be detrimental during long trips in space.

## Figures and Tables

**Table 1:** Skin biochemical parameters

	*Ground*	*Space*
Hydration (%)	58.4±3.4	58.5±2.9
	(μg per mg wet weight)	
Total collagen	293±55	300±54
Newly synthesized procollagen soluble in 0.15 M NaCl	2.2±0.3	3.0±0.6*
1 M NaCl	2.3±0.3	3.4±0.7**
Collagen soluble in 0.5 M HAc	23.2±3.9	24.5±3.2

*P=0.05, **P=0.03, Student's *t*-test.

**Table 2:** Genes involved in extracellular matrix homeostasis significantly modulated (fold change ⩾1.5, *P*⩽0.05) in space (S) versus ground (G) mice

*Gene symbol*	*Gene title*	*Fold change S/G*	P*-value* a
*Structural macromolecules and post-translational enzymes*			
*Col1a1*	Collagen, type I, alpha1	1.53	0.045
*Emilin2*	Elastin microfibril interfacer 2	−2.55	0.029
*Pcolce2*	Procollagen C-endopeptidase enhancer2	−1.72	0.034
*P4ha1*	Procollagen-proline 4-hydroxylase	−1.59	0.010
			
*Matricellular proteins*			
*Tnxb*	Tenascin XB	−1.69	0.017
*Cyr 61/CCN1*	Cysteine rich protein 61	2.84	0.004
*Ctgf/CCN2*	Connective tissue growth factor	2.65	0.001
			
*Hyaluronan synthesis*			
*Has2*	Hyaluronan synthase 2	−1.85	0.031
			
*Dermo-epidermal junction*			
*Col4a4*	Collagen, type IV, alpha 4	−1.72	0.040
*Col7a1*	Collagen, type VII, alpha 1	1.73	0.010
*Lama2*	Laminin, alpha 2	−1.68	0.034
			
*Matrix degradation*			
*Adamts1*	A disintegrin-like and metallopeptidase with thrombospondin type 1 motif, 1	1.57	0.002
*Adamts9*	A disintegrin-like and metallopeptidase with thrombospondin type 1 motif, 9	1.69	0.035
*Plau*	Plasminogen activator, urokinase	−2.19	0.002
*Serpine 1 (PAI1)*	Serine peptidase inhibitor, clade E, member 1	3.96	0.010
			
*Cell–matrix interactions*			
*Itga1*	Integrin alpha 1	−2.08	0.024
*Itgb1*	Integrin beta 1	2.07	0.005
*Itgb1bp2*	Integrin beta 1 binding protein 2	2.57	0.000
*Itgb2*	Integrin beta 2	−2.03	0.047
*Ddr2*	Discoidin domain receptor, member 2	−1.50	0.026

a Student’s *t*-test.

**Table 3:** Expression of hair follicles and interfollicular epidermal keratins

*Gene symbol*	*Gene title*	*Space mice*			*Ground mice*		
		*WT*	*Tg1*	*Tg2*	*WT*	*Tg1*	*Tg2*
*Hair follicles keratins*							
*Krt25*	Keratin 25	30322	33324	19052	37	54017	8177
*Krt26*	Keratin 26	2918	6404	2187	70	12812	524
*Krt27*	Keratin 27	15939	26423	9608	7	41441	3521
*Krt28*	Keratin 28	481	854	229	46	1741	200
*Krt34*	Keratin 34	7177	15010	7473	30	30606	1162
*Krt35*	Keratin 35	4450	3389	2233	7	7172	1197
*Krt71*	Keratin 71	6141	7584	4058	31	15802	1435
*Krt72*	Keratin 72	2967	6535	1820	103	14306	607
*Krt73*	Keratin 73	5845	7741	3476	49	17588	1876
*Krt75*	Keratin 75	3659	4066	2882	1181	6438	746
*Krt81*	Keratin 81	5824	10525	5432	165	21426	1248
*Krt85*	Keratin 85	6156	6109	3377	80	15270	1217
*Krt86*	Keratin 86	4308	9611	4342	5	20312	614
							
*Interfollicular epidermal keratins*							
*Krt1*	Keratin 1	7965	10103	8234	7135	4964	4866
*Krt5*	Keratin 5	12547	12938	10587	16372	12781	10096
*Krt6a*	Keratin 6A	6579	11621	8041	6510	12866	4035
*Krt10*	Keratin 10	50853	49514	49304	53390	39709	45046
*Krt14*	Keratin 14	12435	13312	10087	15079	15938	14753
*Krt15*	Keratin 15	27353	28871	30512	37239	23270	20753
*Krt17*	Keratin 17	135	124	135	106	135	70
*Krt23*	Keratin 23	5231	5238	5340	4221	4357	3203
*Krt77*	Keratin 77	32941	31471	34046	35237	26529	30776
*Krt78*	Keratin 78	1273	1050	1333	1236	659	907
*Krt79*	Keratin 79	8630	6897	6880	7511	3591	7508
*Krt80*	Keratin 80	7576	7254	7754	7735	6785	4424

Abbreviations: Tg, transgenic; WT, wild type.

**Table 4:** Genes with recognized function in muscle atrophy, differentiation, or contraction and significantly regulated in space versus ground groups

*Gene symbol*	*Protein*	*Fold change*	P*-value*
*Contractile fibers*			
*Abra* a	Actin binding rho-activating protein	3.14	0.0046
*Actn*2^a^^,^ b	Actinin alpha 2	3.01	0.0066
*Ankrd23* a	Ankyrin repeat domain 23	3.10	0.0043
*Csrp3* a ^,^ b	Cysteine and glycine-rich protein 3	2.08	0.015
*Des* a ^,^ b	Desmin	2.06	0.0063
*Flnb*	Filamin, beta	2.38	0.015
*Flnc*	Filamin c, gamma	3.12	0.035
*Ldb3* a ^,^ b	Lim domain binding 3	2.42	0.00081
*Lmod2* b	Leiomodin 2 (cardiac)	3.35	0.014
*Mybpc*1	Myosin-binding protein C, slow type	2.23	0.0097
*Mybpc*2^a^	Myosin-binding protein C, fast type	2.23	0.019
*Myh2* a	Myosin, heavy polypeptide 2, skeletal muscle, adult	2.46	0.03
*Mylk2*	Myosin, light polypeptide kinase 2, skeletal muscle	17.51	0.00003
*Myom1* a	Myomesin 1	2.42	0.0064
*Myom2* a ^,^ b	Myomesin 2	3.40	0.0042
*Myom3*	Myomesin, family member 3	2.15	0.027
*Myot* a	Myotilin	2.50	0.002
*Myoz2* a ^,^ b	Myozenin 2	2.78	0.008
*Myoz3*	Myozenin 3	2.20	0.0076
*Neb* a	Nebulin	2.48	0.0065
*Nexn*	Nexilin	2.65	0.0021
*Nrap*	Nebulin-related anchoring protein	2.38	0.012
*Obscn* a	Obscurin, cytoskeletal calmodulin, and titin-interacting rhogef	2.99	0.0071
*Pdlim3* b	PDZ and LIM domain 3	3.71	0.00018
*Pdlim7*	PDZ and LIM domain 7	2.15	0.015
*Sgcg* a	Sarcoglycan, gamma (dystrophin-associated glycoprotein)	2.00	0.011
*Smtnl1*	Smoothelin-like 1 (localization?)	2.26	0.027
*Smyd1* a ^,^ b	SET and MYND domain containing 1	2.38	0.02
*Synm*	Synemin, intermediate filament protein	2.80	0.00023
*Synpo2*	Synaptopodin 2	2.57	0.0035
*Synpo2l* a	Synaptopodin 2 like	2.56	0.0025
*Tcap* b	Titin-cap	2.07	0.0045
*Tmod1*	Tropomodulin 1	2.26	0.0041
*Tmod4* a ^,^ b	Tropomodulin 4	2.37	0.0003
*Tnni2* a	Troponin 1, skeletal, fast	2.14	0.012
*Tpm2* a	Tropomyosin 2, beta	2.03	0.0026
*Trim54*	Tripartite motif-containing 54	2.46	0.0017
*Ttn* a ^,^ b	Titin	2.04	0.018
*Xirp1* a	Xin actin-binding repeat containing 1	2.87	0.042
			
*Sarcoleme, neuromuscular junction, and excitation–contraction coupling*			
*Ank1*	Ankyrin 1, erythroid	2.03	0.0022
*Atp2a1*	Atpase, Ca++ transporting, cardiac muscle, fast twitch 1	2.06	0.016
*Cacna1s*	Calcium channel, voltage-dependent, L type, alpha 1S subunit	2.56	0.0019
*Camk2d*	Calcium/calmodulin-dependent protein kinase II delta	2.22	0.0074
*Casq1* a	Calsequestrin 1	2.50	0.011
*Cav3* a ^,^ b	Caveolin 3	2.26	0.0067
*Hrc* a	histidine-rich calcium binding protein	2.06	0.03
*Itgb1bp2* b	Integrin beta 1-binding protein 2	2.57	0.00067
*Jph2* a ^,^ b	Junctophilin 2	2.30	0.03
*Jsrp1*	Junctional sarcoplasmic reticulum protein 1	2.70	0.0019
*Pacsin3* a	Protein kinase C and casein kinase substrate in neurons 3	2.37	0.00002
*Ryr1*	Ryanodin receptor 1, skeletal muscle	2.42	0.011
*Slc8a3* a	Solute carrier family 8 (sodium/calcium exchanger), member 3	2.24	0.012
*Sypl2*	Synaptophysin-like 2	2.01	0.0071
*Trdn* a	Triadin	2.25	0.003
			
*Oxidative phosphorylation, glycogen breakdown, and glycolysis*			
*Agl*	Amylo-1,6-glucosidase, 4-alpha-glucanotransferase	2.11	0.0057
*Ckmt2* a ^,^ b	Creatine kinase, mitochondrial 2 (sarcomeric)	3.30	0.0002
*Cox6a2* a ^,^ b	Cytochrome *c* oxidase subunit via polypeptide 2	2.13	0.0077
*Eno3* a	Enolase 3, beta muscle	2.49	0.0014
*Mb* a	Myoglobin	3.16	0.00016
*Pfkm*	Phosphofructokinase, muscle	2.52	0.0026
*Pgam2* a ^,^ b	Phosphoglycerate mutase 2	2.94	0.0012
*Phka1*	Phosphorylase kinase alpha 1	2.02	0.0038
*Phkg1*	Phosphorylase kinase gamma 1	2.72	0.013
*Ppp1r3c*	Protein phosphatase 1, regulatory (inhibitor) subunit 3C	2.34	0.009
*Pygm*	Muscle glycogen phosphorylase	2.71	0.0049
*Txnip*	Thioredoxin interacting protein	2.07	0.023
			
*Myogenesis and muscle development, and atrophy*			
*Abra* a	Actin-binding Rho-activating protein	3.14	0.0091
*Alpk3*	Alpha-kinase 3	2.2	0.0039
*Asb2* a	Ankyrin repeat and SOCS box containing 2	3.58	0.0015
*Atf3* a	Activating transcription factor 3	2.00	0.0063
*Capn3* a ^,^ b	Calpain 3	2.03	0.0019
*Csrp3* a ^,^ b	Cysteine and glycine-rich protein 3	2.08	0.015
*Dmrt2*	Doublesex and MAB3-related transcription factor 2	−2.49	0.0027
*Fbxo32* b	F-box protein 32	2.13	0.046
*Hspb7* b	Heat shock 27 kDa protein family, member 7 (cardiovascular)	3.73	0.0052
*Kbtbd10* a	Kelch repeat and BTB (POZ) domain containing 10	3.39	0.00063
*Lbx1*	Ladybird homeobox 1	2.73	0.0005
*Mef2c* a ^,^ b	Myocyte enhancer factor 2C	2.11	0.0015
*Murc*	Muscle-related coiled-coil protein	2.29	0.034
*Myf6* a ^,^ b	Myogenic factor 6	2.22	0.0047
*Myog* a ^,^ b	Myogenin	2.13	0.026
*Pdlim3*	PDZ and LIM domain 3	3.71	0.00018
*Pdlim7*	PDZ and LIM domain 7	2.15	0.015
*Rbm24* a	RNA binding motif 24	3.05	0.0011
*Serpine 1* a	Plasminogen activator inhibitor 1, PAI 1	3.96	0.00989
*Smyd1* a ^,^ b	SET and MYND domain containing 1	2.38	0.02
*Trim63* a ^,^ b	Tripartite motif-containing 63	4.98	0.016
*Vgll2*	Vestigial like 2 (drosophila)	2.23	0.028

Genes recognized as transcriptional targets or having a recognized or potential binding site for

a MEF2 and/or

b myogenin on their promoter.

**Table 5:** Enriched systems in the skin of space mice

	P*-value*
*Molecular function*	
Structural constituent of muscle	1.31E−14
Titin binding	2.31E−07
Cytoskeletal binding	2.32E−07
Actin binding	1.52E−05
Actinin binding	2.08E−04
	
*Biological process*	
Muscle system process	3.73E−18
Muscle contraction	2.63E−16
Striated muscle contraction	6.13E−11
Muscle structure development	2.83E−09
Muscle tissue development	7.37E−09
	
*Cellular component*	
Myofibril	5.07E−33
Contractile fiber	8.10E−33
Sarcomere	1.12E−32
I band	9.52E−24
	
*Mouse phenotype*	
Abnormal muscle contractility	2.71E−06
Impaired skeletal contractility	3.09E−05
Impaired muscle contractility	3.32E−05
Abnormal skeletal muscle morphology	1.05E−04
Abnormal muscle physiology	1.10E−04
	
*Pathway*	
Striated muscle contraction	7.38E−07
Genes involved in striated muscle contraction	9.19E−07
Genes involved in muscle contraction	3.02E−05
Diurnally regulated genes with circadian orthologs	1.46E−03
Genes involved in glycogen breakdown	4.71E−03
	
*Interactions*	
Titin (TTN)	6.05E−09
Titin-cap (TCAP)	1.82E−03
Nebulin (NEB)	3.29E−03
Myozenin 3 (MYOZ3)	4.03E−03
Tripartite motif-containing 63 (TRIM63)	7.51E−03

## Notes

Supplementary Table 1S

Supplementary Table 2S

Supplementary Table 3S

Supplementary Table 4S

Supplementary Table 5S
