# Supplementary material for: Skin physiology in microgravity: a 3-month stay aboard ISS induces dermal atrophy and affects cutaneous muscle and hair follicles cycling in mice
Source: NPJ Microgravity. 2015 May 27;1:15002–. doi: 10.1038/npjmgrav.2015.2 (PMC5515501; doi:10.1038/npjmgrav.2015.2)
Supplement: Supplementary Table 1S [file npjmgrav20152-s1.doc]

**Table 1S: Primers, amplicon size and PCR efficiency (E_PCR_)**

| **Targets** | **Primers 5’ – 3’** | **Amplicon size (bp)** | **E_PCR_** |
| --- | --- | --- | --- |
| *Agl* | Fwd : gagtggctgtggccaattgggta  Rev : tgagctccggaaggcctttccat | 163 | 1.07 |
| *Actb* | Fwd: TTGACATCCGATAAGACCTCTATGC  Rev: TTGCTGATCCACATCTGCTGGAAG | 217 | 0.97 |
| *B2m* | Fwd: TTTCTGGTGCTTGTCTCACTGACC  Rev: TTGGATTTCAATGTGAGGCGGGTG | 150 | 1.01 |
| *Ccnd2* | Fwd: TTCAAGTGCGTGCAGAAGGACATC  Rev: TTCATGGCCAGAGGAAAGACCTCT | 116 | 0.65 |
| *Col1A1* | Fwd: CCCTGAAGTCAGCTGCATACACAA  Rev: CCTACATCTTCTGAGTTTGGTGATAC | 219 | 1.06 |
| *Col1A3* | Fwd: GAGATGTCTGGAAGCCAGAACCAT  Rev: GATCTCCCTTGGGGCCTTGAGGT | 207 | 0.78 |
| *Ctgf* | Fwd: TCTCCACCCGAGTTACCAATGACA  Rev: ACCCCGCAGAACTTAGCCCTGTA | 208 | 0.78 |
| *Cyr61* | Fwd: AGGCAGACCCTGTGAATATAACTC  Rev: ATTGTTTCTCGTTAACTCCACCTC | 289 | 0.68 |
| *Fn* | Fwd: TTACAGAGTAACCACCACTCCCAAA  Rev: GCACTCGATATCCAGTGAGCTGAA | 281 | 0.91 |
| *Gapdh* | Fwd: CCTGGCCAAGGTCATCCATGACA  Rev: GGGATGACCTTGCCCACAGCCTT | 183 | 1.13 |
| *Mef2C* | Fwd : TGCCGCCATCTGCCCTCAGTCA  Rev : CCTCTCGGTCGCTCCCATCGTA | 243/147 | 1.08 |
| *Mmp2* | Fwd: AGATCTTCTTCTTCAAGGACCGGTT  Rev: GGCTGGTCAGTGGCTTGGGGTA | 225 | 0.37 |
| *Mmp3* | Fwd: GATCTCTTCATTTTGGCCATCTCTTC | 246 | 0.54 |
|  | Rev: CTCCAGTATTTGTCCTCTACAAAGAA |  |  |
| *Myct1* | Fwd: TTGGGCTTGCAATCGGAGGATTTC  Rev: GTCCTGTTGAGGCCGTGGTTGTA | 136 | 1.63 |
| *Neb* | Fwd : aacgcctgtcactccagacatgg  Rev : ttccctaacccgcctcaTCTCTG | 145 | 0.84 |
| *Pgam2* | Fwd : ctttgcccttctggaatgaggaga  Rev : ccagtgggcaggttcagctcca | 151 | 0.95 |
| *Ptn* | Fwd : GTAAGATCCCTTGCAACTGGAAGAA  Rev : CTTCTCCTGTTTCTTGCCTTCCTTT | 241 | 1.38 |
| *Pygm* | Fwd : attgtcaatatgctcatgcaccatga  Rev : aggtggctatgttcctgatcacca | 142 | 0.89 |
| *Trdn* | Fwd : aagattccaaagaagcaccaacttca  Rev : aagtacacacattggaagaagctgat | 148 | 1.09 |
| *Ttn* | Fwd : atgacacaactggaaagctcaacca  Rev : gtaggctccccagtaaaggcaca | 146 | 0.87 |
