# Supplementary material for: Skin physiology in microgravity: a 3-month stay aboard ISS induces dermal atrophy and affects cutaneous muscle and hair follicles cycling in mice
Source: NPJ Microgravity. 2015 May 27;1:15002–. doi: 10.1038/npjmgrav.2015.2 (PMC5515501; doi:10.1038/npjmgrav.2015.2)
Supplement: Supplementary Table 2S [file npjmgrav20152-s2.doc]

**Table 2S: Differentially expressed transcripts in space versus ground group (mean fold change >2.0, p-value ≤ 0.05 space vs ground).**

| **Gene symbol** | **Gene title** | **Fold Change** | **p-value** |
| --- | --- | --- | --- |
| *Irs3* | insulin receptor substrate 3 | -7.53 | 0.02963 |
| *A530016L24Rik* | RIKEN cDNA A530016L24 gene | -7.19 | 0.01170 |
| *AW551984* | expressed sequence AW551984 | -6.53 | 0.02154 |
| *Fbxo30* | F-box protein 30 | -5.69 | 0.00041 |
| *Cd209g* | CD209g antigen | -5.15 | 0.04898 |
| *Gm6484* | predicted gene 6484 | -4.36 | 0.03601 |
| *Ccl12* | chemokine (C-C motif) ligand 12 | -4.11 | 0.01098 |
| *Ccl7* | chemokine (C-C motif) ligand 7 | -3.91 | 0.01655 |
| *G0s2* | G0/G1 switch gene 2 | -3.82 | 0.02075 |
| *Myct1* | myc target 1 | -3.62 | 0.02121 |
| *Apex2* | apurinic/apyrimidinic endonuclease 2 | -3.55 | 0.00015 |
| *Pamr1* | peptidase domain containing associated with muscle regeneration 1 | -3.48 | 0.00556 |
| *Akr1c18* | aldo-keto reductase family 1, member C18 | -3.46 | 0.01627 |
| *Mgl1* | macrophage galactose N-acetyl-galactosamine specific lectin 1 | -3.33 | 0.02573 |
| *Cysltr1* | cysteinyl leukotriene receptor 1 | -3.30 | 0.00845 |
| *St3gal1* | ST3 beta-galactoside alpha-2,3-sialyltransferase 1 | -3.28 | 0.02842 |
| *Thap2* | THAP domain containing, apoptosis associated protein 2 | -3.18 | 0.00027 |
| *AI504432* | expressed sequence AI504432 | -3.14 | 0.02316 |
| *AI661384* | expressed sequence AI661384 | -3.05 | 0.01459 |
| *D4Wsu53e* | DNA segment, Chr 4, Wayne State University 53, expressed | -3.02 | 0.03842 |
| *Thrsp* | thyroid hormone responsive SPOT14 homolog (Rattus) | -2.97 | 0.00102 |
| *H2-Q10* | histocompatibility 2, Q region locus 10 | -2.93 | 0.01138 |
| *Cd300lb* | CD300 antigen like family member B | -2.92 | 0.04234 |
| *9630013D21Rik* | RIKEN cDNA 9630013D21 gene | -2.92 | 0.00225 |
| *Agpat2* | 1-acylglycerol-3-phosphate O-acyltransferase 2 | -2.89 | 0.02934 |
| *Slc1a3* | solute carrier family 1, member 3 | -2.86 | 0.04946 |
| *Fcgr1* | Fc receptor, IgG, high affinity I | -2.82 | 0.00610 |
| *2010007H06Rik* | RIKEN cDNA 2010007H06 gene | -2.80 | 0.03966 |
| *Cd48* | CD48 antigen | -2.75 | 0.02247 |
| *Pyhin1* | pyrin and HIN domain family, member 1 | -2.71 | 0.01799 |
| *Rab3il1* | RAB3A interacting protein (rabin3)-like 1 | -2.68 | 0.01169 |
| *Clec4b1* | C-type lectin domain family 4, member b1 | -2.68 | 0.03486 |
| *Cmpk2* | cytidine monophosphate (UMP-CMP) kinase 2, mitochondrial | -2.67 | 0.00909 |
| *Cygb* | cytoglobin | -2.63 | 0.01291 |
| *Gfpt2* | glutamine fructose-6-phosphate transaminase 2 | -2.63 | 0.03140 |
| *Rbm14* | RNA binding motif protein 14 | -2.60 | 0.00804 |
| *Adrb1* | adrenergic receptor, beta 1 | -2.60 | 0.03429 |
| *Irf4* | interferon regulatory factor 4 | -2.57 | 0.04786 |
| *Zfp467* | zinc finger protein 467 | -2.55 | 0.00501 |
| *Hrh1 /// LOC100041871* | histamine receptor H1 /// similar to histamine receptor H1 | -2.54 | 0.00004 |
| *Rbm12b* | RNA binding motif protein 12B | -2.54 | 0.00044 |
| *Enpep* | glutamyl aminopeptidase | -2.54 | 0.00621 |
| *Gm5870 /// Sec61b* | Sec61 beta subunit pseudogene /// Sec61 beta subunit | -2.52 | 0.03406 |
| *Zfp189* | zinc finger protein 189 | -2.52 | 0.00596 |
| *Tlr4* | toll-like receptor 4 | -2.50 | 0.01117 |
| *C5ar1* | complement component 5a receptor 1 | -2.49 | 0.04575 |
| *Dmrt2* | doublesex and mab-3 related transcription factor 2 | -2.49 | 0.00271 |
| *Tgtp /// Tgtp2* | T-cell specific GTPase /// T-cell specific GTPase 2 | -2.49 | 0.00035 |
| *Gm4979* | predicted gene 4979 | -2.46 | 0.00454 |
| *Zfp429* | zinc finger protein 429 | -2.45 | 0.00056 |
| *Rbp4* | retinol binding protein 4, plasma | -2.44 | 0.03057 |
| *9530028C05* | hypothetical protein 9530028C05 | -2.44 | 0.02255 |
| *Sc5d* | sterol-C5-desaturase (fungal ERG3, delta-5-desaturase) homolog | -2.44 | 0.00218 |
| *Gbp6* | guanylate binding protein 6 | -2.43 | 0.00152 |
| *Ptgfr* | prostaglandin F receptor | -2.39 | 0.03225 |
| *Gbp3* | guanylate binding protein 3 | -2.38 | 0.00935 |
| *Agpat9* | 1-acylglycerol-3-phosphate O-acyltransferase 9 | -2.38 | 0.04829 |
| *Irgm2* | immunity-related GTPase family M member 2 | -2.38 | 0.00087 |
| *Trim36* | tripartite motif-containing 36 | -2.37 | 0.01939 |
| *Pla2g2d* | phospholipase A2, group IID | -2.36 | 0.02279 |
| *Sfpq* | splicing factor proline/glutamine rich | -2.36 | 0.00530 |
| *LOC623121* | similar to Interferon-activatable protein 203 (Ifi-203) | -2.33 | 0.02090 |
| *Samd9l* | sterile alpha motif domain containing 9-like | -2.33 | 0.01388 |
| *Glipr1* | GLI pathogenesis-related 1 (glioma) | -2.33 | 0.03429 |
| *Rtp4* | receptor transporter protein 4 | -2.32 | 0.01963 |
| *2700050L05Rik* | RIKEN cDNA 2700050L05 gene | -2.32 | 0.01511 |
| *BB182297* | expressed sequence BB182297 | -2.32 | 0.00070 |
| *Mmd* | monocyte to macrophage differentiation-associated | -2.31 | 0.04934 |
| *Zfp420* | zinc finger protein 420 | -2.30 | 0.01454 |
| *Banp* | BTG3 associated nuclear protein | -2.30 | 0.04710 |
| *Oasl2* | 2'-5' oligoadenylate synthetase-like 2 | -2.29 | 0.04073 |
| *Prokr1* | prokineticin receptor 1 | -2.29 | 0.00228 |
| *Gvin1* | GTPase, very large interferon inducible 1 | -2.27 | 0.00867 |
| *Diras2* | DIRAS family, GTP-binding RAS-like 2 | -2.27 | 0.00304 |
| *Zc4h2* | zinc finger, C4H2 domain containing | -2.27 | 0.00579 |
| *Zfp418* | zinc finger protein 418 | -2.27 | 0.00108 |
| *Acvr1c* | activin A receptor, type IC | -2.26 | 0.04681 |
| *Acaca* | acetyl-Coenzyme A carboxylase alpha | -2.26 | 0.03703 |
| *Zbp1* | Z-DNA binding protein 1 | -2.25 | 0.00021 |
| *Ccnd2* | cyclin D2 | -2.25 | 0.01294 |
| *Ints8* | integrator complex subunit 8 | -2.25 | 0.00891 |
| *5330426P16Rik* | RIKEN cDNA 5330426P16 gene | -2.24 | 0.00752 |
| *Ubxn2b* | UBX domain protein 2B | -2.24 | 0.02810 |
| *Fam46c* | family with sequence similarity 46, member C | -2.23 | 0.01648 |
| *Hoxb6* | homeo box B6 | -2.22 | 0.01612 |
| *Fam111a* | family with sequence similarity 111, member A | -2.22 | 0.02445 |
| *Vwa5a* | von Willebrand factor A domain containing 5A | -2.22 | 0.01361 |
| *Cxcl12* | chemokine (C-X-C motif) ligand 12 | -2.21 | 0.00083 |
| *Pilra* | paired immunoglobin-like type 2 receptor alpha | -2.21 | 0.01413 |
| *Cd68* | CD68 antigen | -2.19 | 0.04866 |
| *Plau* | plasminogen activator, urokinase | -2.19 | 0.00353 |
| *Ankrd37* | ankyrin repeat domain 37 | -2.19 | 0.01899 |
| *LOC100048346 /// Usp18* | similar to ubiquitin specific protease UBP43 /// ubiquitin specific peptidase 18 | -2.18 | 0.04105 |
| *Tspan4* | tetraspanin 4 | -2.18 | 0.01960 |
| *Cfp* | complement factor properdin | -2.17 | 0.04039 |
| *Slc27a1* | solute carrier family 27 (fatty acid transporter), member 1 | -2.16 | 0.01790 |
| *Ifi47* | interferon gamma inducible protein 47 | -2.16 | 0.00204 |
| *Zfp719* | zinc finger protein 719 | -2.16 | 0.00738 |
| *Gbp2* | guanylate binding protein 2 | -2.15 | 0.00027 |
| *Prr16* | proline rich 16 | -2.14 | 0.02880 |
| *St8sia4* | ST8 alpha-N-acetyl-neuraminide alpha-2,8-sialyltransferase 4 | -2.14 | 0.02024 |
| *Rsl1 /// Zfp429 /// Zfp455 /// Zfp456* | regulator of sex limited protein 1 /// zinc finger protein 429/455/456 | -2.14 | 0.00843 |
| *Nck1* | non-catalytic region of tyrosine kinase adaptor protein 1 | -2.13 | 0.00089 |
| *Tmem120b* | transmembrane protein 120B | -2.13 | 0.03755 |
| *Iigp1* | interferon inducible GTPase 1 | -2.12 | 0.00238 |
| *Fasn* | fatty acid synthase | -2.12 | 0.00232 |
| *Batf3* | basic leucine zipper transcription factor, ATF-like 3 | -2.12 | 0.04184 |
| *1190005F20Rik* | RIKEN cDNA 1190005F20 gene | -2.12 | 0.00320 |
| *4921525H12Rik* | RIKEN cDNA 4921525H12 gene | -2.11 | 0.01508 |
| *Ptpro* | protein tyrosine phosphatase, receptor type, O | -2.11 | 0.02517 |
| *Zfp354b* | zinc finger protein 354B | -2.11 | 0.00897 |
| *Pygl* | liver glycogen phosphorylase | -2.10 | 0.02746 |
| *3300001P08Rik* | RIKEN cDNA 3300001P08 gene | -2.10 | 0.00011 |
| *2810055F11Rik* | RIKEN cDNA 2810055F11 gene | -2.10 | 0.04504 |
| *C3* | complement component 3 | -2.09 | 0.01428 |
| *Chtf8* | CTF8, chromosome transmission fidelity factor 8 homolog | -2.09 | 0.00911 |
| *Galnt12* | UDP-N-acetyl-alpha-D-galactosamine:polypeptide N-acetylgalactosaminyltransferase 12 | -2.09 | 0.00889 |
| *Itga1* | integrin alpha 1 | -2.08 | 0.04897 |
| *LOC100048559 /// Sfrs1* | similar to splicing factor, arginine/serine-rich 1 (ASF/SF2) | -2.08 | 0.03420 |
| *Gm7899 /// Hspa8 /// LOC624853* | predicted gene 7899 /// heat shock protein 8 | -2.08 | 0.00442 |
| *Ccdc112* | coiled-coil domain containing 112 | -2.07 | 0.00370 |
| *Purg* | purine-rich element binding protein G | -2.07 | 0.00107 |
| *Ang* | angiogenin, ribonuclease, RNase A family, 5 | -2.07 | 0.00700 |
| *Kcns3* | potassium voltage-gated channel, delayed-rectifier, subfamily S, member 3 | -2.07 | 0.02531 |
| *Mcpt4* | mast cell protease 4 | -2.06 | 0.03581 |
| *Klhdc5* | kelch domain containing 5 | -2.06 | 0.00023 |
| *Esr1* | estrogen receptor 1 (alpha) | -2.05 | 0.02839 |
| *Fzd7* | frizzled homolog 7 (Drosophila) | -2.05 | 0.02025 |
| *Mitd1* | MIT, microtubule interacting and transport, domain containing 1 | -2.04 | 0.00427 |
| *Nrarp* | Notch-regulated ankyrin repeat protein | -2.04 | 0.03927 |
| *Tbpl1* | TATA box binding protein-like 1 | -2.04 | 0.01845 |
| *Kctd21* | potassium channel tetramerisation domain containing 21 | -2.03 | 0.00530 |
| *Lyl1* | lymphoblastomic leukemia 1 | -2.03 | 0.02479 |
| *Zfp207* | zinc finger protein 207 | -2.02 | 0.00289 |
| *9430012M22Rik* | RIKEN cDNA 9430012M22 gene | -2.02 | 0.04596 |
| *Baz1b* | bromodomain adjacent to zinc finger domain, 1B | -2.02 | 0.00364 |
| *Zik1* | zinc finger protein interacting with K protein 1 | -2.01 | 0.01981 |
| *AI844685* | expressed sequence AI844685 | -2.00 | 0.00159 |
| *Rap2a* | RAS related protein 2a | -2.00 | 0.00366 |
| *B230354K17Rik* | RIKEN cDNA B230354K17 gene | -2.00 | 0.00005 |
| *Chordc1* | cysteine and histidine-rich domain (CHORD)-containing, zinc-binding protein 1 | -2.00 | 0.02512 |
| *Tlr2* | toll-like receptor 2 | -2.00 | 0.02615 |
| *D630030B22Rik* | RIKEN cDNA D630030B22 gene | 2.00 | 0.04901 |
| *Atf3* | activating transcription factor 3 | 2.00 | 0.00630 |
| *Hhatl* | hedgehog acyltransferase-like | 2.00 | 0.03970 |
| *Atl2* | atlastin GTPase 2 | 2.00 | 0.00635 |
| *6330512M04Rik* | RIKEN cDNA 6330512M04 gene | 2.00 | 0.01652 |
| *Kera* | keratocan | 2.00 | 0.02328 |
| *Sypl2* | synaptophysin-like 2 | 2.01 | 0.00708 |
| *Ankrd9* | ankyrin repeat domain 9 | 2.02 | 0.03494 |
| *Rapgef4* | Rap guanine nucleotide exchange factor (GEF) 4 | 2.02 | 0.00245 |
| *Rtn2* | reticulon 2 (Z-band associated protein) | 2.02 | 0.02492 |
| *2310002L09Rik* | RIKEN cDNA 2310002L09 gene | 2.02 | 0.00933 |
| *Capn3* | calpain 3 | 2.03 | 0.00195 |
| *Phka1* | phosphorylase kinase alpha 1 | 2.03 | 0.00384 |
| *Ank1* | ankyrin 1, erythroid | 2.03 | 0.00222 |
| *2310040G24Rik* | RIKEN cDNA 2310040G24 gene | 2.03 | 0.01439 |
| *2310038E17Rik* | RIKEN cDNA 2310038E17 gene | 2.03 | 0.00860 |
| *Rragd* | Ras-related GTP binding D | 2.04 | 0.00755 |
| *6720463M24Rik* | RIKEN cDNA 6720463M24 gene | 2.04 | 0.00002 |
| *Sgcg* | sarcoglycan, gamma (dystrophin-associated glycoprotein) | 2.04 | 0.02479 |
| *Gm10567* | predicted gene 10567 | 2.04 | 0.02848 |
| *Slc25a12* | solute carrier family 25 (mitochondrial carrier, Aralar), member 12 | 2.04 | 0.00818 |
| *D1Ertd564e* | DNA segment, Chr 1, ERATO Doi 564, expressed | 2.05 | 0.00063 |
| *Spsb1* | splA/ryanodine receptor domain and SOCS box containing 1 | 2.05 | 0.01972 |
| *Cabc1* | chaperone, ABC1 activity of bc1 complex like (S, pombe) | 2.05 | 0.00894 |
| *Slc25a33* | solute carrier family 25, member 33 | 2.06 | 0.02307 |
| *Des* | desmin | 2.06 | 0.00630 |
| *LOC100047619 /// Slc7a5* | similar to solute carrier family 7, member 5 | 2.06 | 0.02322 |
| *Hrc* | histidine rich calcium binding protein | 2.06 | 0.03012 |
| *Atp2a1* | ATPase, Ca++ transporting, cardiac muscle, fast twitch 1 | 2.06 | 0.01589 |
| *5730411F24Rik* | RIKEN cDNA 5730411F24 gene | 2.06 | 0.00985 |
| *C920006O11Rik* | RIKEN cDNA C920006O11 gene | 2.06 | 0.00288 |
| *Mef2c* | myocyte enhancer factor 2C | 2.06 | 0.00150 |
| *Schip1* | schwannomin interacting protein 1 | 2.07 | 0.00909 |
| *Zfp628* | zinc finger protein 628 | 2.07 | 0.00287 |
| *Txnip* | thioredoxin interacting protein | 2.07 | 0.02287 |
| *Tcap* | titin-cap | 2.07 | 0.00455 |
| *Hfe2* | hemochromatosis type 2 (juvenile) (human homolog) | 2.08 | 0.01280 |
| *B4galnt2* | beta-1,4-N-acetyl-galactosaminyl transferase 2 | 2.08 | 0.04435 |
| *Csrp3* | cysteine and glycine-rich protein 3 | 2.08 | 0.01523 |
| *Smr2* | submaxillary gland androgen regulated protein 2 | 2.08 | 0.02871 |
| *Rad18* | RAD18 homolog (S, cerevisiae) | 2.09 | 0.02511 |
| *2810403D21Rik* | RIKEN cDNA 2810403D21 gene | 2.09 | 0.00284 |
| *Gm13238* | predicted gene 13238 | 2.10 | 0.00356 |
| *Stc1* | stanniocalcin 1 | 2.11 | 0.02464 |
| *Agl* | amylo-1,6-glucosidase, 4-alpha-glucanotransferase | 2.11 | 0.00574 |
| *Cyp2r1* | cytochrome P450, family 2, subfamily r, polypeptide 1 | 2.11 | 0.03599 |
| *Neurl1a* | neuralized homolog 1A (Drosophila) | 2.11 | 0.00857 |
| *Npas2* | neuronal PAS domain protein 2 | 2.12 | 0.01730 |
| *Usp54* | ubiquitin specific peptidase 54 | 2.12 | 0.04157 |
| *1810032O08Rik* | RIKEN cDNA 1810032O08 gene | 2.12 | 0.03523 |
| *1110020C03Rik* | RIKEN cDNA 1110020C03 gene | 2.13 | 0.00425 |
| *Myog* | myogenin | 2.13 | 0.02623 |
| *Fbxo32* | F-box protein 32 | 2.13 | 0.04595 |
| *Cox6a2* | cytochrome c oxidase, subunit VI a, polypeptide 2 | 2.13 | 0.00767 |
| *9030203C11Rik* | RIKEN cDNA 9030203C11 gene | 2.14 | 0.01036 |
| *Adh6a* | alcohol dehydrogenase 6A (class V) | 2.14 | 0.02108 |
| *Rdh1* | retinol dehydrogenase 1 (all trans) | 2.15 | 0.02111 |
| *LOC100039111* | Similar to cadherin 11 | 2.15 | 0.01486 |
| *Myom3* | myomesin family, member 3 | 2.15 | 0.02715 |
| *Pdlim7* | PDZ and LIM domain 7 | 2.15 | 0.01525 |
| *AU019559* | expressed sequence AU019559 | 2.15 | 0.00954 |
| *Slc9a8* | solute carrier family 9 (sodium/hydrogen exchanger), member 8 | 2.15 | 0.04080 |
| *Slc25a26* | solute carrier family 25 (mitochondrial carrier, phosphate carrier), member 26 | 2.16 | 0.02272 |
| *Popdc2* | popeye domain containing 2 | 2.16 | 0.00637 |
| *Ces8* | carboxylesterase 8 (putative) | 2.16 | 0.04859 |
| *Atad4* | ATPase family, AAA domain containing 4 | 2.16 | 0.02243 |
| *Arrdc3* | arrestin domain containing 3 | 2.17 | 0.00563 |
| *Psmd9* | proteasome (prosome, macropain) 26S subunit, non-ATPase, 9 | 2.17 | 0.03226 |
| *Kbtbd5* | kelch repeat and BTB (POZ) domain containing 5 | 2.17 | 0.02036 |
| *Duxbl* | double homeobox B-like | 2.17 | 0.00622 |
| *Yrdc* | yrdC domain containing (E,coli) | 2.17 | 0.00244 |
| *AB041803* | cDNA sequence AB041803 | 2.17 | 0.01297 |
| *3021401C12Rik* | RIKEN cDNA 3021401C12 gene | 2.19 | 0.00025 |
| *Chkb-cpt1b /// Cpt1b* | choline kinase beta, carnitine palmitoyltransferase 1b, muscle transcription unit | 2.19 | 0.00992 |
| *Nos1* | nitric oxide synthase 1, neuronal | 2.19 | 0.00655 |
| *Alpk3* | alpha-kinase 3 | 2.20 | 0.00394 |
| *Myoz3* | myozenin 3 | 2.20 | 0.00758 |
| *Hist1h1c* | histone cluster 1, H1c | 2.20 | 0.01062 |
| *Nr4a1* | nuclear receptor subfamily 4, group A, member 1 | 2.20 | 0.01597 |
| *Errfi1* | ERBB receptor feedback inhibitor 1 | 2.21 | 0.02216 |
| *Ltb4r1* | leukotriene B4 receptor 1 | 2.22 | 0.02016 |
| *Camk2d* | calcium/calmodulin-dependent protein kinase II, delta | 2.22 | 0.00740 |
| *Myf6* | myogenic factor 6 | 2.22 | 0.00457 |
| *A230058F20Rik* | RIKEN cDNA A230058F20 gene | 2.22 | 0.01149 |
| *Vgll2* | vestigial like 2 homolog (Drosophila) | 2.23 | 0.02805 |
| *A830039N20Rik* | RIKEN cDNA A830039N20 gene | 2.23 | 0.00773 |
| *2410002O22Rik /// LOC100044479* | RIKEN cDNA 2410002O22 gene | 2.23 | 0.00045 |
| *Mybpc2* | myosin binding protein C, fast-type | 2.23 | 0.01933 |
| *9430034N14Rik* | RIKEN cDNA 9430034N14 gene | 2.23 | 0.04202 |
| *BB114814* | expressed sequence BB114814 | 2.23 | 0.03111 |
| *Slc10a6* | solute carrier family 10 (sodium/bile acid cotransporter family), member 6 | 2.23 | 0.01713 |
| *Slc8a3* | solute carrier family 8 (sodium/calcium exchanger), member 3 | 2.24 | 0.01202 |
| *Eif2ak1* | eukaryotic translation initiation factor 2 alpha kinase 1 | 2.24 | 0.00509 |
| *7120451J01Rik* | RIKEN cDNA 7120451J01 gene | 2.25 | 0.00968 |
| *1110002E22Rik* | RIKEN cDNA 1110002E22 gene | 2.25 | 0.00476 |
| *2310015B20Rik* | RIKEN cDNA 2310015B20 gene | 2.25 | 0.00469 |
| *Amhr2* | anti-Mullerian hormone type 2 receptor | 2.25 | 0.01522 |
| *Gm5785 /// LOC100047799* | predicted gene 5785 /// similar to TAF4A RNA polymerase II | 2.25 | 0.00020 |
| *Smtnl1* | smoothelin-like 1 | 2.26 | 0.02717 |
| *Tmod1* | tropomodulin 1 | 2.26 | 0.00407 |
| *Pla2g4f* | phospholipase A2, group IVF | 2.26 | 0.01283 |
| *Cav3* | caveolin 3 | 2.26 | 0.00670 |
| *Fam107a* | family with sequence similarity 107, member A | 2.26 | 0.04182 |
| *Trdn* | triadin | 2.26 | 0.00966 |
| *Mpp3* | membrane protein, palmitoylated 3 | 2.27 | 0.00836 |
| *A130004G07Rik* | RIKEN cDNA A130004G07 gene | 2.27 | 0.00572 |
| *Zc3h6* | zinc finger CCCH type containing 6 | 2.27 | 0.01850 |
| *Cap2* | CAP, adenylate cyclase-associated protein, 2 (yeast) | 2.27 | 0.00664 |
| *Ndrg2* | N-myc downstream regulated gene 2 | 2.28 | 0.00071 |
| *Car14* | carbonic anhydrase 14 | 2.28 | 0.02135 |
| *A2bp1* | ataxin 2 binding protein 1 | 2.28 | 0.01007 |
| *Rad54b* | RAD54 homolog B (S, cerevisiae) | 2.29 | 0.00898 |
| *Bxdc5* | brix domain containing 5 | 2.29 | 0.00223 |
| *Cirbp* | cold inducible RNA binding protein | 2.29 | 0.03618 |
| *9430098F02Rik* | RIKEN cDNA 9430098F02 gene | 2.29 | 0.02835 |
| *Smtnl2* | smoothelin-like 2 | 2.29 | 0.01003 |
| *Murc* | muscle-related coiled-coil protein | 2.29 | 0.03388 |
| *Elp4* | elongation protein 4 homolog (S, cerevisiae) | 2.30 | 0.00385 |
| *Jph2* | junctophilin 2 | 2.30 | 0.02997 |
| *Ttn* | titin | 2.30 | 0.01054 |
| *0610033M10Rik* | RIKEN cDNA 0610033M10 gene | 2.31 | 0.02445 |
| *Arhgef10l* | Rho guanine nucleotide exchange factor (GEF) 10-like | 2.32 | 0.03853 |
| *Asb4* | ankyrin repeat and SOCS box-containing 4 | 2.32 | 0.01033 |
| *Abcb4* | ATP-binding cassette, sub-family B (MDR/TAP), member 4 | 2.32 | 0.00231 |
| *Tmem38a* | transmembrane protein 38A | 2.33 | 0.00096 |
| *Amot* | angiomotin | 2.33 | 0.00121 |
| *Slc38a4* | solute carrier family 38, member 4 | 2.34 | 0.00496 |
| *Ppapdc3* | phosphatidic acid phosphatase type 2 domain containing 3 | 2.34 | 0.02743 |
| *Atr* | Ataxia telangiectasia and Rad3 related | 2.34 | 0.01367 |
| *Mfsd2* | major facilitator superfamily domain containing 2 | 2.34 | 0.03353 |
| *Ppp1r3c* | protein phosphatase 1, regulatory (inhibitor) subunit 3C | 2.34 | 0.00905 |
| *AI452195* | expressed sequence AI452195 | 2.35 | 0.00086 |
| *Slc27a2* | solute carrier family 27 (fatty acid transporter), member 2 | 2.35 | 0.02013 |
| *LOC100046468* | hypothetical protein LOC100046468 | 2.35 | 0.02775 |
| *Asb12* | ankyrin repeat and SOCS box-containing 12 | 2.36 | 0.00144 |
| *Cyr61* | cysteine rich protein 61 | 2.37 | 0.00181 |
| *Tmod4* | tropomodulin 4 | 2.37 | 0.00030 |
| *Pacsin3* | protein kinase C and casein kinase substrate in neurons 3 | 2.37 | 0.00002 |
| *Synm* | synemin, intermediate filament protein | 2.37 | 0.00275 |
| *Smyd1* | SET and MYND domain containing 1 | 2.37 | 0.01953 |
| *Flnb* | Filamin, beta | 2.38 | 0.01518 |
| *Padi2* | peptidyl arginine deiminase, type II | 2.38 | 0.01065 |
| *Lrrc2* | leucine rich repeat containing 2 | 2.39 | 0.00259 |
| *Cand2* | cullin-associated and neddylation-dissociated 2 (putative) | 2.39 | 0.00859 |
| *Ldb3* | LIM domain binding 3 | 2.40 | 0.00174 |
| *5430417C01Rik* | RIKEN cDNA 5430417C01 gene | 2.40 | 0.01516 |
| *Serpinb3b /// Serpinb3c* | serine (or cysteine) peptidase inhibitor, clade B (ovalbumin), member 3B | 2.40 | 0.00861 |
| *Sec14l5* | SEC14-like 5 (S, cerevisiae) | 2.41 | 0.00628 |
| *Agxt2l1* | alanine-glyoxylate aminotransferase 2-like 1 | 2.41 | 0.00487 |
| *Myom1* | myomesin 1 | 2.42 | 0.00643 |
| *Shisa4* | shisa homolog 4 (Xenopus laevis) | 2.42 | 0.00384 |
| *Ryr1* | ryanodine receptor 1, skeletal muscle | 2.43 | 0.01129 |
| *Trp53inp1* | transformation related protein 53 inducible nuclear protein 1 | 2.43 | 0.02647 |
| *Camk2b* | calcium/calmodulin-dependent protein kinase II, beta | 2.45 | 0.01752 |
| *Lysmd1* | LysM, putative peptidoglycan-binding, domain containing 1 | 2.45 | 0.00761 |
| *Trim54* | tripartite motif-containing 54 | 2.46 | 0.00166 |
| *Sgk1* | serum/glucocorticoid regulated kinase 1 | 2.46 | 0.00113 |
| *Scn4b* | sodium channel, type IV, beta | 2.47 | 0.00247 |
| *5730416O20Rik* | RIKEN cDNA 5730416O20 gene | 2.47 | 0.00340 |
| *LOC100047138 /// Tesc* | similar to Tescalcin /// tescalcin | 2.47 | 0.00460 |
| *Me3* | malic enzyme 3, NADP(+)-dependent, mitochondrial | 2.48 | 0.00116 |
| *Neb* | nebulin | 2.48 | 0.00650 |
| *Pdss1* | prenyl (solanesyl) diphosphate synthase, subunit 1 | 2.48 | 0.00105 |
| *Cmya5* | cardiomyopathy associated 5 | 2.48 | 0.00585 |
| *Txlnb* | taxilin beta | 2.49 | 0.02892 |
| *D17Ertd663e* | DNA segment, Chr 17, ERATO Doi 663, expressed | 2.49 | 0.03805 |
| *B130052P14Rik* | RIKEN cDNA B130052P14 gene | 2.49 | 0.01840 |
| *Cox7a1* | cytochrome c oxidase, subunit VIIa 1 | 2.49 | 0.00086 |
| *Eno3* | enolase 3, beta muscle | 2.49 | 0.00139 |
| *Casq1* | calsequestrin 1 | 2.50 | 0.01058 |
| *Myot* | myotilin | 2.50 | 0.00199 |
| *Gadd45g* | growth arrest and DNA-damage-inducible 45 gamma | 2.50 | 0.01239 |
| *4833416J08Rik* | RIKEN cDNA 4833416J08 gene | 2.50 | 0.00317 |
| *Fitm1* | fat storage-inducing transmembrane protein 1 | 2.51 | 0.00747 |
| *LOC100047682 /// Phkg1* | similar to phosphorylase kinase, gamma-subunit /// phosphorylase kinase gamma 1 | 2.51 | 0.01565 |
| *Pfkm* | phosphofructokinase, muscle | 2.52 | 0.00263 |
| *Klf9* | Kruppel-like factor 9 | 2.52 | 0.01033 |
| *Mt1* | metallothionein 1 | 2.53 | 0.01335 |
| *Mapt* | microtubule-associated protein tau | 2.54 | 0.00147 |
| *4632433K11Rik* | RIKEN cDNA 4632433K11 gene | 2.55 | 0.03861 |
| *Epha1* | Eph receptor A1 | 2.55 | 0.00385 |
| *B230117O15Rik* | RIKEN cDNA B230117O15 gene | 2.56 | 0.00257 |
| *Synpo2l* | synaptopodin 2-like | 2.56 | 0.00245 |
| *Cacna1s* | calcium channel, voltage-dependent, L type, alpha 1S subunit | 2.56 | 0.00191 |
| *H19* | H19 fetal liver mRNA | 2.56 | 0.03185 |
| *Defb2* | defensin beta 2 | 2.56 | 0.03676 |
| *Lcn2* | lipocalin 2 | 2.57 | 0.03759 |
| *Synpo2* | synaptopodin 2 | 2.57 | 0.00351 |
| *Itgb1bp2* | integrin beta 1 binding protein 2 | 2.57 | 0.00066 |
| *Mt2* | metallothionein 2 | 2.57 | 0.00074 |
| *2310010M20Rik* | RIKEN cDNA 2310010M20 gene | 2.57 | 0.04741 |
| *Il20* | interleukin 20 | 2.58 | 0.02658 |
| *6230414M07Rik* | RIKEN cDNA 6230414M07 gene | 2.58 | 0.04364 |
| *Aox4* | aldehyde oxidase 4 | 2.58 | 0.00840 |
| *Mybpc1* | myosin binding protein C, slow-type | 2.60 | 0.01102 |
| *Tbk1* | TANK-binding kinase 1 | 2.61 | 0.03206 |
| *Atxn7l1* | ataxin 7-like 1 | 2.63 | 0.02011 |
| *Nupl2* | nucleoporin like 2 | 2.63 | 0.00577 |
| *Srl* | sarcalumenin | 2.65 | 0.00155 |
| *Nexn* | nexilin | 2.65 | 0.00212 |
| *Ctgf* | connective tissue growth factor | 2.65 | 0.00177 |
| *Rcan2* | regulator of calcineurin 2 | 2.66 | 0.00022 |
| *Ucp3* | uncoupling protein 3 (mitochondrial, proton carrier) | 2.66 | 0.01429 |
| *1200003I10Rik /// 1200015M12Rik* | RIKEN cDNA 1200003I10 gene | 2.66 | 0.00218 |
| *A630005I04Rik* | RIKEN cDNA A630005I04 gene | 2.66 | 0.00815 |
| *Jsrp1* | junctional sarcoplasmic reticulum protein 1 | 2.70 | 0.00189 |
| *Pygm* | muscle glycogen phosphorylase | 2.71 | 0.00493 |
| *BC042782* | cDNA sequence BC042782 | 2.72 | 0.00394 |
| *Adm* | adrenomedullin | 2.72 | 0.03630 |
| *Kcnq5* | potassium voltage-gated channel, subfamily Q, member 5 | 2.72 | 0.00939 |
| *Phkg1* | phosphorylase kinase gamma 1 | 2.72 | 0.01275 |
| *LOC100046232 /// Nfil3* | similar to NFIL3/E4BP4 transcription factor /// nuclear factor, interleukin 3, regulated | 2.73 | 0.01539 |
| *Lbx1* | ladybird homeobox homolog 1 (Drosophila) | 2.73 | 0.00050 |
| *Asb15* | ankyrin repeat and SOCS box-containing 15 | 2.74 | 0.00140 |
| *Rapgefl1* | Rap guanine nucleotide exchange factor (GEF)-like 1 | 2.74 | 0.01663 |
| *Arl4d* | ADP-ribosylation factor-like 4D | 2.77 | 0.00988 |
| *Sdr42e1* | short chain dehydrogenase/reductase family 42E, member 1 | 2.78 | 0.00191 |
| *Myoz2* | myozenin 2 | 2.78 | 0.00794 |
| *Adssl1* | adenylosuccinate synthetase like 1 | 2.79 | 0.00154 |
| *Arrdc2* | arrestin domain containing 2 | 2.79 | 0.00166 |
| *Fabp3* | fatty acid binding protein 3, muscle and heart | 2.79 | 0.01934 |
| *Elovl3* | elongation of very long chain fatty acids (FEN1/Elo2, SUR4/Elo3, yeast)-like 3 | 2.80 | 0.00035 |
| *Snrpn /// Snurf* | small nuclear ribonucleoprotein N /// SNRPN upstream reading frame | 2.80 | 0.00327 |
| *Rpl3l* | ribosomal protein L3-like | 2.82 | 0.00399 |
| *Serpinb6c* | serine (or cysteine) peptidase inhibitor, clade B, member 6c | 2.82 | 0.02100 |
| *Cul2* | cullin 2 | 2.82 | 0.00235 |
| *Xirp1* | xin actin-binding repeat containing 1 | 2.87 | 0.04221 |
| *AU014973* | expressed sequence AU014973 | 2.89 | 0.01758 |
| *Sh3bgr* | SH3-binding domain glutamic acid-rich protein | 2.90 | 0.00072 |
| *Slc26a3* | solute carrier family 26, member 3 | 2.90 | 0.01140 |
| *Ppm1j* | protein phosphatase 1J | 2.90 | 0.00347 |
| *Hemgn* | hemogen | 2.91 | 0.01689 |
| *Plin5* | perilipin 5 | 2.94 | 0.02799 |
| *Pgam2* | phosphoglycerate mutase 2 | 2.94 | 0.00116 |
| *1700056N10Rik* | RIKEN cDNA 1700056N10 gene | 2.94 | 0.01561 |
| *Hspb6* | heat shock protein, alpha-crystallin-related, B6 | 2.96 | 0.00329 |
| *Coro6* | coronin 6 | 2.98 | 0.00389 |
| *Obscn* | obscurin, cytoskeletal calmodulin and titin-interacting RhoGEF | 2.99 | 0.00707 |
| *Kcnc1* | potassium voltage gated channel, Shaw-related subfamily, member 1 | 3.01 | 0.01804 |
| *Actn2* | actinin alpha 2 | 3.01 | 0.00665 |
| *1810029B16Rik* | RIKEN cDNA 1810029B16 gene | 3.03 | 0.02736 |
| *Hmgcr* | 3-hydroxy-3-methylglutaryl-Coenzyme A reductase | 3.04 | 0.00002 |
| *Gm3336* | predicted gene 3336 | 3.04 | 0.02986 |
| *Rbm24* | RNA binding motif protein 24 | 3.05 | 0.00112 |
| *Ankrd23* | ankyrin repeat domain 23 | 3.10 | 0.00427 |
| *Flnc* | filamin C, gamma | 3.12 | 0.03493 |
| *Abra* | actin-binding Rho activating protein | 3.14 | 0.00915 |
| *Mb* | myoglobin | 3.16 | 0.00016 |
| *E030010A14Rik* | RIKEN cDNA E030010A14 gene | 3.18 | 0.01090 |
| *2310016C08Rik* | RIKEN cDNA 2310016C08 gene | 3.20 | 0.00020 |
| *Slc47a1* | solute carrier family 47, member 1 | 3.23 | 0.00419 |
| *Akr1c21* | aldo-keto reductase family 1, member C21 | 3.24 | 0.00245 |
| *Gm6377* | predicted gene 6377 | 3.24 | 0.04746 |
| *Rprd2* | regulation of nuclear pre-mRNA domain containing 2 | 3.27 | 0.00129 |
| *Usp32* | ubiquitin specific peptidase 32 | 3.28 | 0.00199 |
| *Asb10* | ankyrin repeat and SOCS box-containing 10 | 3.28 | 0.01327 |
| *Ankrd2* | ankyrin repeat domain 2 (stretch responsive muscle) | 3.29 | 0.00566 |
| *Ckmt2* | creatine kinase, mitochondrial 2 | 3.30 | 0.00020 |
| *4432414F05Rik* | RIKEN cDNA 4432414F05 gene | 3.31 | 0.00302 |
| *Slco2a1* | Solute carrier organic anion transporter family, member 2a1 | 3.34 | 0.01097 |
| *Rdh18* | retinol dehydrogenase 18 | 3.34 | 0.02612 |
| *Lmod2* | leiomodin 2 (cardiac) | 3.35 | 0.01419 |
| *6030422H21Rik* | RIKEN cDNA 6030422H21 gene | 3.37 | 0.00361 |
| *Kbtbd10* | kelch repeat and BTB (POZ) domain containing 10 | 3.39 | 0.00063 |
| *Myom2* | myomesin 2 | 3.40 | 0.00042 |
| *Hif3a /// LOC641092* | hypoxia inducible factor 3, alpha subunit | 3.43 | 0.04269 |
| *Asb2* | ankyrin repeat and SOCS box-containing 2 | 3.58 | 0.00154 |
| *Pdlim3* | PDZ and LIM domain 3 | 3.71 | 0.00018 |
| *Hspb7* | heat shock protein family, member 7 (cardiovascular) | 3.73 | 0.00522 |
| *Mansc1* | MANSC domain containing 1 | 3.80 | 0.00273 |
| *Pla2g4e* | phospholipase A2, group IVE | 3.85 | 0.00008 |
| *LOC100047199 /// Odf2* | similar to outer dense fiber of sperm tails 2 /// outer dense fiber of sperm tails 2 | 3.89 | 0.00468 |
| *Serpine1* | serine (or cysteine) peptidase inhibitor, clade E, member 1 | 3.96 | 0.00989 |
| *Klhl30* | kelch-like 30 (Drosophila) | 4.02 | 0.01641 |
| *Apold1* | apolipoprotein L domain containing 1 | 4.03 | 0.00075 |
| *Scn2a1* | sodium channel, voltage-gated, type II, alpha 1 | 4.05 | 0.01655 |
| *Tal2* | T-cell acute lymphocytic leukemia 2 | 4.16 | 0.01505 |
| *Pdk4* | pyruvate dehydrogenase kinase, isoenzyme 4 | 4.17 | 0.00203 |
| *Tbx4* | T-box 4 | 4.40 | 0.01018 |
| *Hspa1l* | heat shock protein 1-like | 4.45 | 0.00155 |
| *1200016E24Rik* | RIKEN cDNA 1200016E24 gene | 4.60 | 0.00276 |
| *Serpinb12* | serine (or cysteine) peptidase inhibitor, clade B (ovalbumin), member 12 | 4.70 | 0.00046 |
| *4833418N02Rik* | RIKEN cDNA 4833418N02 gene | 4.72 | 0.00439 |
| *Trim63* | tripartite motif-containing 63 | 4.98 | 0.01586 |
| *Trp53* | Transformation related protein 53 | 4.98 | 0.00401 |
| *Sgpp2* | sphingosine-1-phosphate phosphotase 2 | 5.17 | 0.00246 |
| *6030439D06Rik* | RIKEN cDNA 6030439D06 gene | 6.00 | 0.00178 |
| *Acot5* | acyl-CoA thioesterase 5 | 6.39 | 0.00176 |
| *Gm13295 /// Gm2169* | predicted gene 13295///2169///2277///2565///2563///3430///3682///3707///3885 | 6.64 | 0.00228 |
| *Ddit4* | DNA-damage-inducible transcript 4 | 6.73 | 0.00874 |
| *Cpamd8 /// Mug-ps1 /// Mug1* | C3 and PZP-like, alpha-2-macroglobulin domain containing 8 /// murinoglobulin, pseudogene 1 | 8.14 | 0.02787 |
| *Mylk2* | myosin, light polypeptide kinase 2, skeletal muscle | 17.51 | 0.00003 |
