# Supplementary material for: Skin physiology in microgravity: a 3-month stay aboard ISS induces dermal atrophy and affects cutaneous muscle and hair follicles cycling in mice
Source: NPJ Microgravity. 2015 May 27;1:15002–. doi: 10.1038/npjmgrav.2015.2 (PMC5515501; doi:10.1038/npjmgrav.2015.2)
Supplement: Supplementary Table 3S [file npjmgrav20152-s3.doc]

**Table 3S: Comparison between the mean fold changes of selected genes expression in Space versus Ground group as measured by microarray and RT-qPCR.**

| **Gene symbol** | **µArray**  **S/G** | **qPCR/GAPDH-βActin-B2M**  **S/G** |
| --- | --- | --- |
| ***Agl*** | 2.11 | 1.81 |
| ***Ccnd2*** | -1.64 | -1.52 |
| ***Col1a1*** | 1.53 | 1.98 |
| ***Col3a1*** | 1.09 | 2.34 |
| ***Ctgf*** | 2.65 | 2.21 |
| ***Cyr61*** | 2.37 | 2.40 |
| ***Fn*** | -1.68 | 2.55 |
| ***Mef2c*** | 2.19 | 1.95 |
| ***Mmp2*** | -1.32 | -1.07 |
| ***Mmp3*** | 1.02 | 1.08 |
| ***Myct1*** | -3.62 | -3.79 |
| ***Neb*** | 2.48 | 2.44 |
| ***Pgam2*** | 2.94 | 2.41 |
| ***Ptn*** | 1.08 | -1.15 |
| ***Pygm*** | 2.71 | 2.00 |
| ***Trdn*** | 2.26 | 2.18 |
| ***Ttn*** | 2.30 | 2.17 |

Pearson’s correlation coefficient: r= 0.78, slope=0.75
