# Supplementary material for: Skin physiology in microgravity: a 3-month stay aboard ISS induces dermal atrophy and affects cutaneous muscle and hair follicles cycling in mice
Source: NPJ Microgravity. 2015 May 27;1:15002–. doi: 10.1038/npjmgrav.2015.2 (PMC5515501; doi:10.1038/npjmgrav.2015.2)
Supplement: Supplementary Table 4S [file npjmgrav20152-s4.doc]

**Table 4S: Differentially expressed genes (absolute fold change ≥ 2.0 and p-value ≤ 0.05) between space (S) and ground (G)** groups containing a potential or recognized binding site for MEF2. Genes related to muscle metabolism are in bold.

| **Gene Symbol** | **Gene Title** | **Fold change (S/G)** | **P-Value** |
| --- | --- | --- | --- |
| ***Ankrd23*** | ankyrin repeat domain 23 | 3.10 | 0.0043 |
| *Arrdc3* | arrestin domain containing 3 | 2.17 | 0.0056 |
| *Asb2* | ankyrin repeat and SOCS box-containing 2 | 3.58 | 0.0015 |
| *Asb4* | ankyrin repeat and SOCS box-containing 4 | 2.32 | 0.010 |
| *Asb15* | ankyrin repeat and SOCS box-containing 15 | 2.74 | 0.014 |
| *Asph* | aspartate-beta-hydroxylase | 3.03 | 0.0012 |
| ***Atf3*** | activating transcription factor 3 | 2.00 | 0.0063 |
| *Atl2* | atlastin GTPase 2 | 2.00 | 0.0063 |
| *Banp* | BTG3 associated nuclear protein | -2.30 | 0.047 |
| ***Capn3*** | calpain 3 | 2.03 | 0.0019 |
| ***Casq1*** | calsequestrin 1 | 2.50 | 0.011 |
| ***Cav3*** | caveolin 3 | 2.26 | 0.0067 |
| ***Cktm2*** | creatine kinase, mitochondrial 2 | 3.30 | 0.0002 |
| ***Cox6a2*** | cytochrome c oxidase, subunit VI a, polypeptide 2 | 2.13 | 0.0077 |
| *Cpt1b* | carnitine palmitoyltransferase 1b, muscle | 2.19 | 0.00496 |
| ***Csrp3*** | cysteine and glycine-rich protein 3 | 2.08 | 0.015 |
| ***Ctgf*** | connective tissue growth factor | 2.65 | 0.0018 |
| ***Des*** | Desmin | 2.06 | 0.0063 |
| ***Eno3*** | enolase 3, beta muscle | 2.49 | 0.0014 |
| *Esr1* | estrogen receptor 1 (alpha) | -2.05 | 0.028 |
| *Fam134b* | family with sequence similarity 134, member B | 2.04 | 0.0045 |
| *Fitm1* | fat storage-inducing transmembrane protein 1 | 2.51 | 0.0075 |
| *Hbp1* | high mobility group box transcription factor 1 | 3.34 | 0.014 |
| *Hfe2* | hairy and enhancer of split 1 (Drosophila) | 2.08 | 0.013 |
| *Hist1h1c* | histone cluster 1, H1c | 2.34 | 0.0014 |
| *Hoxb6* | homeo box B6 | -2.22 | 0.016 |
| *Hrc* | histidine rich calcium binding protein | 2.06 | 0.030 |
| *Ip6k2* | inositol hexaphosphate kinase 2 | 2.01 | 0.0054 |
| *Kbtbd5* | kelch repeat and BTB (POZ) domain containing 5 | 2.17 | 0.020 |
| ***Kbtbd10*** | kelch repeat and BTB (POZ) domain containing 10 | 3.39 | 0.00063 |
| *Kcnn1* | potassium intermediate/small conductance calcium-activated channel, subfamily N, member 1 | 3.01 | 0.018 |
| *Kcnq5* | potassium voltage-gated channel, subfamily Q, member 5 | 2.72 | 0.0094 |
| ***Mb*** | Myoglobin | 3.16 | 0.00016 |
| ***Mef2c*** | myocyte enhancer factor 2C | 2.06 | 0.0012 |
| ***Mybpc2*** | myosin binding protein C, fast-type | 2.23 | 0.019 |
| ***Myf6*** | myogenic factor 6 | 2.22 | 0.0046 |
| ***Myog*** | Myogenin | 2.13 | 0.026 |
| ***Myom2*** | myomesin 2 | 3.69 | 0.00025 |
| ***Myoz2*** | myozenin 2 | 2.78 | 0.0079 |
| *Ndrg2* | N-myc downstream regulated gene 2 | 2.78 | 0.00071 |
| ***Neb*** | nebulin | 2.48 | 0.0065 |
| *Nfil3* | nuclear factor, interleukin 3, regulated | 2.73 | 0.0077 |
| *Nr4a1* | nuclear receptor subfamily 4, group A, member 1 | 2.20 | 0.016 |
| ***Obscn*** | obscurin, cytoskeletal calmodulin and titin-interacting RhoGEF | 2.99 | 0.0071 |
| ***Pacsin3*** | protein kinase C and casein kinase substrate in neurons 3 | 2.48 | 0.00002 |
| ***Pgam2*** | phosphoglycerate mutase 2 | 2.94 | 0.0012 |
| *Phka2* | phosphorylase kinase alpha 2 | 2.04 | 0.042 |
| *Ptpro* | protein tyrosine phosphatase, receptor type, O | -2.11 | 0.025 |
| *Scn4b* | sodium channel, type IV, beta | 2.47 | 0.024 |
| ***Sgcg*** | sarcoglycan, gamma (dystrophin-associated glycoprotein) | 2.00 | 0.011 |
| ***Slc8a3*** | solute carrier family 8 (sodium/calcium exchanger), member 3 | 2.24 | 0.012 |
| *Slc25a4* | solute carrier family 25 (mitochondrial carrier, adenine nucleotide translocator), member 4 | 2.02 | 0.0003 |
| *Slco2a1* | Solute carrier organic anion transporter family, member 2a1 | 3.34 | 0.011 |
| ***Smyd1*** | SET and MYND domain containing 1 | 2.37 | 0.020 |
| *Sox5* | SRY-box containing gene 5 | 2.11 | 0.040 |
| *Stc1* | stanniocalcin 1 | 2.11 | 0.025 |
| ***Synpo2l*** | synaptopodin 2-like | 2.56 | 0.0024 |
| *Tlk2* | tousled-like kinase 2 (Arabidopsis) | 2.25 | 0.010 |
| ***Tmod4*** | tropomodulin 4 | 2.37 | 0.0003 |
| ***Tnni2*** | troponin I, skeletal, fast 2 | 2.14 | 0.012 |
| ***Tpm2*** | tropomyosin 2, beta | 2.24 | 0.042 |
| *Tpp2* | tripeptidyl peptidase II | 2.30 | 0.0026 |
| ***Trdn*** | triadin | 2.26 | 0.0048 |
| ***Ttn*** | titin | 2.30 | 0.010 |
| *Wac* | WW domain containing adaptor with coiled-coil | 2.06 | 0.011 |
| ***Xirp1*** | xin actin-binding repeat containing 1 | 2.87 | 0.042 |
