# Supplementary material for: Skin physiology in microgravity: a 3-month stay aboard ISS induces dermal atrophy and affects cutaneous muscle and hair follicles cycling in mice
Source: NPJ Microgravity. 2015 May 27;1:15002–. doi: 10.1038/npjmgrav.2015.2 (PMC5515501; doi:10.1038/npjmgrav.2015.2)
Supplement: Supplementary Table 5S [file npjmgrav20152-s5.doc]

**Table 5S: Expressed genes (> 100 arbitrary units) involved in extracellular matrix homeostasis. Fold change between space vs ground group. Genes with a fold change ≥ 1.5 and a p-value ≤ 0.05 (S vs G) are highlighted.**

| **Gene Symbol** | | **Gene Title** | **Fold change**  **(S/G)** | **p-Value** |
| --- | --- | --- | --- | --- |
| **Structural macromolecules** | | | |  |
| ***Col1a1*** | collagen, type I, alpha 1 | | 1.53 | 0.04465 |
| *Col1a2* | collagen, type I, alpha 2 | | 1.19 | 0.17425 |
| *Col3a1* | collagen, type III, alpha 1 | | 1.09 | 0.20792 |
| *Col5a1* | collagen, type V, alpha 1 | | -1.12 | 0.36452 |
| *Col5a2* | collagen, type V, alpha 2 | | -1.04 | 0.44821 |
| *Col5a3* | collagen, type V, alpha 3 | | -1.64 | 0.10779 |
| *Col12a1* | collagen, type XII, alpha 1 | | -1.08 | 0.34698 |
| *Col14a1* | collagen, type XIV, alpha 1 | | -1.56 | 0.09572 |
| *Fn1* | fibronectin 1 | | -1.68 | 0.07965 |
| *Vtn* | vitronectin | | 1.22 | 0.16219 |
| **Elasticfibers** | | |  |  |
| *Eln* | elastin | | 1.09 | 0.37970 |
| *Fbn1* | fibrillin 1 | | -1.47 | 0.13589 |
| *Fbn2* | fibrillin 2 | | 1.54 | 0.15447 |
| *Fbln1* | fibulin 1 | | -1.31 | 0.02809 |
| *Fbln2* | fibulin 2 | | -1.49 | 0.06280 |
| *Fbln5* | fibulin 5 | | -1.16 | 0.18581 |
| *Emilin1* | elastin microfibrilinterfacer 1 | | -1.16 | 0.34030 |
| ***Emilin2*** | elastin microfibrilinterfacer 2 | | -2.55 | 0.02851 |
| *Mfap5* | microfibrillarassociatedprotein 5 | | -1.82 | 0.11260 |
| *Mfap1a* | microfibrillar-associatedprotein 1A | | -1.13 | 0.01874 |
| *Mfap2* | microfibrillar-associatedprotein 2 | | 1.17 | 0.04197 |
| *Mfap3* | microfibrillar-associatedprotein 3 | | -1.27 | 0.00978 |
| *Mfap3l* | microfibrillar-associatedprotein 3-like | | -1.11 | 0.28581 |
| *Mfap4* | microfibrillar-associatedprotein 4 | | -1.24 | 0.27604 |
| *Ltbp1* | latent transforming growth factor beta binding protein 1 | | -1.24 | 0.24437 |
| *Ltbp2* | latent transforming growth factor beta binding protein 2 | | 1.03 | 0.47067 |
| *Ltbp3* | latent transforming growth factor beta binding protein 3 | | -1.29 | 0.06610 |
| *Ltbp4* | latent transforming growth factor beta binding protein 4 | | -1.11 | 0.19869 |
| **Post-translational enzymes** | | |  |  |
| *Bmp1* | Bone morphogeneticprotein 1 | | -1.19 | 0.23737 |
| ***Pcolce2*** | procollagen C-endopeptidaseenhancer 2 | | -1.72 | 0.03424 |
| *Pcolce1* | procollagen C-endopeptidaseenhancerprotein | | 1.05 | 0.44252 |
| *Adamts2* | a disintegrin-like and metallopeptidase (reprolysin type) with thrombospondin type 1 motif, 2 | | -1.18 | 0.32591 |
| *Plod1* | procollagen-lysine, 2-oxoglutarate 5-dioxygenase 1 | | -1.28 | 0.24282 |
| *Plod2* | procollagen lysine, 2-oxoglutarate 5-dioxygenase 2 | | -1.44 | 0.23676 |
| *Plod3* | procollagen-lysine, 2-oxoglutarate 5-dioxygenase 3 | | -1.22 | 0.09646 |
| ***P4ha1*** | procollagen-proline, 2-oxoglutarate 4-dioxygenase (proline 4-hydroxylase), alpha 1 polypeptide | | -1.59 | 0.01013 |
| *P4ha2* | procollagen-proline, 2-oxoglutarate 4-dioxygenase (proline 4-hydroxylase), alpha II polypeptide | | -1.19 | 0.28762 |
| *Lox* | lysyloxidase | | 1.10 | 0.37934 |
| *Loxl1* | lysyloxidase-like 1 | | -1.86 | 0.06993 |
| *Loxl2* | lysyloxidase-like 2 | | -1.48 | 0.17470 |
| *Loxl3* | lysyloxidase-like 3 | | -1.25 | 0.22749 |
| *Loxl4* | lysyloxidase-like 4 | | -1.06 | 0.42093 |
| **Matricellularproteins** | | |  |  |
| *Thbs1* | thrombospondin 1 | | -1.21 | 0.36037 |
| *Thbs2* | thrombospondin 2 | | -1.48 | 0.02346 |
| *Thbs3* | thrombospondin 3 | | -1.24 | 0.12914 |
| *Thbs4* | thrombospondin 4 | | -1.09 | 0.26997 |
| *Spock2* | sparc/osteonectin | | 1.25 | 0.19357 |
| *Tnc* | tenascin C | | 1.00 | 0.49870 |
| ***Tnxb*** | tenascin XB | | -1.69 | 0.01724 |
| ***Cyr61*** | cysteinerichprotein 61 | | 2.84 | 0.00373 |
| ***Ctgf*** | connective tissue growth factor | | 2.65 | 0.00089 |
| *Nov* | nephroblastomaoverexpressedgene | | -1.89 | 0.06729 |
| **Hyaluronicacid and hyalectans** | | |  |  |
| ***Has2*** | hyaluronansynthase 2 | | -1.85 | 0.03052 |
| *Vcan* | versican | | -1.20 | 0.27125 |
| *Hmmr* | hyaluronan mediated motility receptor (RHAMM) | | -1.38 | 0.17685 |
| *CD44* | CD 44 antigen | | -1.19 | 0.04104 |
| **Dermo-epidermaljunction** | | |  |  |
| *Agrn* | agrin | | 1.09 | 0.19448 |
| *Col4a2* | collagen, type IV, alpha 2 | | -1.08 | 0.36399 |
| *Col4a3bp* | collagen, type IV, alpha 3 | | 1.01 | 0.46388 |
| ***Col4a4*** | collagen, type IV, alpha 4 | | -1.72 | 0.04022 |
| *Col4a5* | collagen, type IV, alpha 5 | | -1.02 | 0.42440 |
| *Col4a6* | collagen, type IV, alpha 6 | | 1.03 | 0.45871 |
| ***Col7a1*** | collagen, type VII, alpha 1 | | 1.73 | 0.01040 |
| *Col17a1* | collagen, type XVII, alpha 1 | | 1.04 | 0.43355 |
| *Col23a1* | collagen, type XXIII, alpha 1 | | 1.50 | 0.04860 |
| *Lamb1-1* | laminin B1 subunit 1 | | -1.49 | 0.01751 |
| *Lamc3* | laminin gamma 3 | | -1.07 | 0.41887 |
| ***Lama2*** | laminin, alpha 2 | | -1.68 | 0.03372 |
| *Lama3* | laminin, alpha 3 | | -1.01 | 0.48400 |
| *Lama4* | laminin, alpha 4 | | -1.84 | 0.10142 |
| *Lama5* | laminin, alpha 5 | | 1.40 | 0.01181 |
| *Lamb2* | laminin, beta 2 | | -1.23 | 0.12536 |
| *Lamb3* | laminin, beta 3 | | 1.01 | 0.47799 |
| *Lamc1* | laminin, gamma 1 | | -1.09 | 0.23254 |
| *Lamc2* | laminin, gamma 2 | | -1.03 | 0.42254 |
| *perlecan* | Hspg2 | | -1.13 | 0.35693 |
| *Nid1* | nidogen 1 | | -1.44 | 0.12695 |
| *Nid2* | nidogen 2 | | -1.45 | 0.00009 |
| *Agrn* | agrin | | 1.09 | 0.19448 |
| **SLRP** | | |  |  |
| *Fmod* | fibromodulin | | 1.41 | 0.09574 |
| *Dcn* | decorin | | -1.05 | 0.21665 |
| *Bgn* | biglycan | | 1.06 | 0.36061 |
| ***Lum*** | lumican | | 1.40 | 0.00896 |
| *Dpt* | dermatopontin | | -1.22 | 0.17418 |
| **Matrix degradation enzymes and regulators** | | |  |  |
| *Mmp2* | matrix metallopeptidase 2 | | -1.32 | 0.08853 |
| *Mmp3* | matrix metallopeptidase 3 | | 1.02 | 0.47501 |
| *Mmp9* | matrix metallopeptidase 9 | | -1.10 | 0.34980 |
| *Mmp11* | matrix metallopeptidase 11 | | -1.73 | 0.12197 |
| *Mmp12* | matrix metallopeptidase 12 | | -1.76 | 0.14239 |
| *Mmp14* | matrix metallopeptidase 14 | | -1.23 | 0.26717 |
| *Mmp15* | matrix metallopeptidase 15 | | 1.45 | 0.03810 |
| *Mmp23* | matrix metallopeptidase 23 | | -1.09 | 0.36753 |
| ***Adamts1*** | a disintegrin-like and metallopeptidase with thrombospondin type 1 motif, 1 | | 1.57 | 0.00151 |
| *Adamts5* | a disintegrin-like and metallopeptidase with thrombospondin type 1 motif, 5 (aggrecanase-2) | | -1.48 | 0.15263 |
| ***Adamts9*** | a disintegrin-like and metallopeptidase with thrombospondin type 1 motif, 9 | | 1.69 | 0.03512 |
| *Adamts10* | a disintegrin-like and metallopeptidase with thrombospondin type 1 motif, 10 | | -1.31 | 0.18084 |
| *Adamts12* | a disintegrin-like and metallopeptidase with thrombospondin type 1 motif, 12 | | -1.37 | 0.07590 |
| *Adamts15* | a disintegrin-like and metallopeptidase with thrombospondin type 1 motif, 15 | | 1.37 | 0.11266 |
| *Adamts16* | a disintegrin-like and metallopeptidase  with thrombospondin type 1 motif, 16 | | -1.39 | 0.24633 |
| *Timp1* | tissue inhibitor of metalloproteinase 1 | | -1.19 | 0.33247 |
| *Timp2* | tissue inhibitor of metalloproteinase 2 | | -1.30 | 0.06143 |
| *Timp3* | tissue inhibitor of metalloproteinase 3 | | -1.42 | 0.00520 |
| *Timp4* | tissue inhibitor of metalloproteinase 4 | | -1.46 | 0.22126 |
| *Plat* | plasminogenactivator, tissue | | -1.28 | 0.12255 |
| ***Plau*** | plasminogenactivator, urokinase | | -2.19 | 0.00176 |
| *Plaur* | plasminogenactivator, urokinase receptor | | 1.24 | 0.08692 |
| ***Serpine1*** | Serine (cysteine) peptidase inhibitor, clade E, member 1 (Plasminogen Activtor Inhibitor-1, PAI-1) | | 3.96 | 0.00989 |
| **Cell -matrix interactions** | | |  |  |
| ***Itga1*** | integrin alpha 1 | | -2.08 | 0.02448 |
| *Itga2b* | integrin alpha 2b | | 1.37 | 0.15618 |
| *Itga3* | integrin alpha 3 | | 1.26 | 0.09467 |
| *Itga4* | integrin alpha 4 | | -1.46 | 0.16292 |
| *Itga6* | integrin alpha 6 | | -1.06 | 0.30173 |
| *Itga7* | integrin alpha 7 | | -1.17 | 0.18207 |
| *Itga8* | integrin alpha 8 | | -1.01 | 0.47988 |
| *Itga9* | integrin alpha 9 | | -1.35 | 0.03174 |
| *Itgae* | integrin alpha E, epithelial-associated | | -1.04 | 0.41744 |
| *Itfg1* | integrin alpha FG-GAP repeat containing 1 | | -1.11 | 0.06935 |
| *Itfg2* | integrin alpha FG-GAP repeat containing 2 | | 1.07 | 0.19425 |
| *Itfg3* | integrin alpha FG-GAP repeat containing 3 | | 1.09 | 0.24101 |
| *Itgal* | integrin alpha L | | -1.23 | 0.09348 |
| *Itgav* | integrin alpha V | | -1.12 | 0.04447 |
| *Itgax* | integrin alpha X | | -1.17 | 0.24543 |
| *Itgb1* | integrin beta 1 (fibronectin receptor beta) | | -1.17 | 0.16708 |
| ***Itgb1*** | integrin beta 1 (fibronectin receptor beta) | | 2.07 | 0.00512 |
| *Itgb1bp1* | integrin beta 1 binding protein 1 ICAP-1 | | -1.19 | 0.09341 |
| ***Itgb1bp2*** | integrin beta 1 bindingprotein 2 | | 2.57 | 0.00033 |
| ***Itgb2*** | integrin beta 2 | | -2.03 | 0.04672 |
| *Itgb4* | integrin beta 4 | | -1.09 | 0.38013 |
| *Itgb5* | integrin beta 5 | | -1.16 | 0.02039 |
| *Itgb7* | integrin beta 7 | | -1.40 | 0.11793 |
| *Ilk* | integrinlinked kinase | | -1.39 | 0.03539 |
| *Itgbl1* | integrin, beta-like 1 | | -1.34 | 0.15110 |
| *Ddr1* | discoidin domain receptor family, member 1 | | 1.12 | 0.21453 |
| ***Ddr2*** | discoidin domain receptor family, member 2 | | -1.50 | 0.02596 |
| **Others** | | |  |  |
| *Col6a1* | collagen, type VI, alpha 1 | | -1.18 | 0.23150 |
| *Col6a2* | collagen, type VI, alpha 2 | | -1.03 | 0.45718 |
| *Col6a3* | collagen, type VI, alpha 3 | | -1.32 | 0.20771 |
| ***Col8a1*** | collagen, type VIII, alpha 1 | | 1.54 | 0.02915 |
| *Col8a2* | collagen, type VIII, alpha 2 | | 1.16 | 0.35531 |
| *Col11a1* | collagen, type XI, alpha 1 | | -1.20 | 0.32428 |
| *Col15a1* | collagen, type XV, alpha 1 | | -1.15 | 0.08370 |
| *Col16a1* | collagen, type XVI, alpha 1 | | -1.05 | 0.40611 |
| *Col18a1* | collagen, type XVIII, alpha 1 | | -1.08 | 0.27208 |
